# Oxidized Carbon Nanoparticles Enhance Cellular Energetics With Application to Injured Brain

**DOI:** 10.1002/adhm.202401629

**Published:** 2024-09-27

**Authors:** Karthik Mouli, Anton V. Liopo, Emily A. McHugh, Erica Underwood, Jing Zhao, Pramod K. Dash, Anh T. T. Vo, Vikas H. Malojirao, Muralidhar L. Hegde, James M. Tour, Paul J. Derry, Thomas A. Kent

**Affiliations:** ^1^ Center for Genomics and Precision Medicine Department of Translational Medicine Institute of Biosciences and Technology Texas A&M Health Science Center Houston TX 77030 USA; ^2^ Department of Chemistry Rice University Houston TX 77005 USA; ^3^ Smalley‐Curl Institute Rice University Houston TX 77005 USA; ^4^ Department of Neurobiology and Anatomy The University of TX McGovern Medical School Houston TX 77030 USA; ^5^ Center for Neuroregeneration Department of Neurosurgery Division of DNA Repair Research Houston Methodist Research Institute Houston TX 77030 USA; ^6^ Department of Neurosciences Weill Cornell Medical College New York NY USA; ^7^ EnMed School of Engineering Medicine Texas A&M University Houston 77030 USA; ^8^ Welch Institute for Advanced Materials Rice University Houston TX 77005 USA; ^9^ The NanoCarbon Center Rice University Houston TX 77005 USA; ^10^ Stanley H. Appel Department of Neurology Houston Methodist Hospital and Research Institute Houston TX 77030 USA

**Keywords:** bioenergetics, lactate, Mitochondria, Oxidized carbon nanozyme, traumatic brain injury

## Abstract

Pro‐energetic effects of functionalized, oxidized carbon nanozymes (OCNs) are reported. OCNs, derived from harsh acid oxidation of single‐wall carbon nanotubes or activated charcoal are previously shown to possess multiple nanozymatic activities including mimicking superoxide dismutase and catalyzing the oxidation of reduced nicotinamide adenine dinucleotide (NADH) to NAD^+^. These actions are predicted to generate a glycolytic shift and enhance mitochondrial energetics under impaired conditions. Impaired mitochondrial energy metabolism is increasingly recognized as an important facet of traumatic brain injury (TBI) pathophysiology and decreases the efficiency of electron transport chain (ETC)‐coupled adenosine triphosphate (ATP) and NAD^+^ regeneration. In vitro, OCNs promote a pro‐aerobic shift in energy metabolism that persists through ETC inhibition and enhances glycolytic flux, glycolytic ATP production, and cellular generation of lactate, a crucial auxiliary substrate for energy metabolism. To address specific mechanisms of iron injury from hemorrhage, OCNs with the iron chelator, deferoxamine (DEF), covalently‐linked were synthesized. DEF‐linked OCNs induce a glycolytic shift in‐vitro and in‐vivo in tissue sections from a rat model of TBI complicated by hemorrhagic contusion. OCNs further reduced hemorrhage volumes 3 days following TBI. These results suggest OCNs are promising as pleiotropic mediators of cell and tissue resilience to injury.

## Graphical abstract

Oxidized carbon nanozymes (OCNs) that catalyze the dismutation of superoxide radical and oxidation of reduced nicotinamide adenine dinucleotide (NADH) to NAD^+^ promote glycolytic and mitochondrial energy metabolism under impaired conditions observed in traumatic brain injury with hemorrhage. OCNs linked with the iron chelator deferoxamine reduce hemorrhage volumes in vivo, suggesting their promise as pleiotropic mediators of resilience to injury.

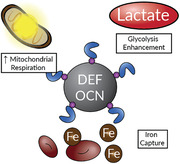

## 1 Introduction

Cellular homeostasis requires the capacity of the cell to maintain energetic stability under a wide range of environmental conditions. The limits of this capacity are reached under pathological conditions such as traumatic brain injuries (TBI), stroke, and sepsis that impair mitochondrial energy metabolism,^[^ ^1^, ^2^, ^3^, ^4^ ^]^ decreasing the ability of cells to harvest energy from metabolic substrates. Under these circumstances as well as a result of the aging process, there is leakage of electrons from complexes I, II and III to diatomic oxygen which decreases the efficiency of electron transport chain (ETC)‐coupled ATP generation and increases the generation of reactive oxygen species (ROS) as a toxic byproduct.^[^ ^3^, ^4^ ^]^ Decreased mitochondrial ETC efficiency compromises the capacity to regenerate oxidized nicotinamide adenine dinucleotide (NAD^+^), an electron carrier required by catabolic pathways such as glycolysis as well as homeostatic mechanisms that reduce the harmful effects of oxidative stress including oxidative DNA damage that requires PARP (poly (ADP‐ribose) polymerase)‐mediated repair.^[^ ^1^, ^5^, ^6^ ^]^ A decreased [NAD^+^]/[NADH] ratio as a result of decreased mitochondrial metabolic efficiency is recognized as a key driver of accelerated cellular aging.^[^ ^3^, ^5^, ^6^ ^]^ Therefore, pharmacological interventions that restore the ability to reconvert NADH to NAD^+^ may increase cellular resilience to stressors that decrease the efficiency of energy metabolic pathways.^[^ ^7^ ^]^

Our group has developed promising neuroprotectants derived from the harsh oxidation of carbon‐rich sources. There has been considerable recent interest in efficient carbon‐based catalysts with an emphasis on their applications to a wide range of biologically‐relevant electron transfer reactions, such as those that use oxygen as a final electron acceptor.^[^ ^8^, ^9^ ^]^ Within the context of brain trauma, nanoscale carbon catalysts – nanozymes – have the added benefit of traversing the blood‐brain barrier, allowing for the targeted treatment of pathologies such as brain tumors, neurodegenerative diseases, and traumatic brain injury.^[^ ^10^ ^]^ Other nanozymes, such as functionalized cerium oxide nanoparticles also attracted attention due to their protective antioxidative effects in models of traumatic brain injury.^[^ ^11^ ^]^ However, ceria nanoparticles exhibit cytotoxicity and genotoxicity in a wide range of cell lines with long‐term exposure,^[^ ^12^ ^]^ potentially limiting their use as a therapeutic agent. In contrast, carbon nanomaterials can be flexibly modified to decrease their in vivo toxicities^[^ ^13^ ^]^ and mediate protection against ROS‐mediated DNA damage.^[^ ^14^ ^]^ Our prototypical carbon nanozyme is the poly(ethylene glycol)‐functionalized hydrophilic carbon cluster (PEG‐HCC) derived from the oleum‐fuming HNO_3_ oxidation of single‐walled carbon nanotubes yielding 3 × 40 nm nanoparticles decorated with carboxyl (─COOH), carbonyl (C═O), and hydroxyl (─OH) functional groups that are later functionalized with poly(ethylene glycol) to improve their circulation time. We showed these particles to be potent superoxide dismutase (SOD) mimetics^[^ ^15^, ^16^ ^]^ that can catalytically transfer electrons,^[^ ^4^ ^]^ which can potentially be used in the treatment of mild traumatic brain injury complicated by hypotensive shock, known to worsens outcome when left untreated,^[^ ^17^, ^18^ ^]^ as well as hemorrhagic or ischemic stroke.^[^ ^14^, ^19^ ^]^ More recently, we developed more clinically translatable nanoparticles derived from medicinal grade activated charcoal (PEG‐cOACs), providing an inexpensive and medicinal grade starting material in contrast to the proprietary single wall carbon nanotubes that served as the precursor for the PEG‐HCC. Optimized acid oxidation of both sources yields biocompatible oxidized carbon nanozymes (OCNs) with a broad redox potential and catalytic SOD mimetic activity,^[^ ^4^, ^16^, ^17^, ^19^ ^]^ These OCNs colocalize with mitochondria in cultured cells and oxidize NADH to reduce ferricytochrome c, a protein that transports electrons between mitochondrial Complexes III and IV.^[^ ^4^ ^]^

The ability of OCNs to function as electron transport nanozymes that catalytically transfer electrons from NADH to electron acceptors (ferricytochrome c, resazurin)^[^ ^4^ ^]^ suggests that they may modulate metabolic pathways which depend on the availability of oxidized electron carriers such as NAD^+^. This action on NADH is supported by the ability of PEG‐HCCs to protect against the mitochondrial ETC complex poisons‐ cyanide and antimycin A.^[^ ^4^ ^]^ Therefore, glycolytic energy metabolism provides a compelling investigative avenue due to its relationship with mitochondrial energy metabolism. While an important feeder pathway into the mitochondria under normal circumstances, glycolysis becomes the primary pathway of cellular ATP generation when mitochondrial function is impaired^1^. Under such circumstances, the oxidation  of NADH to NAD^+^ is essential to ensure the continuation of glycolytic reactions in the cytoplasm.^[^ ^1^ ^]^ We thus hypothesized that by enhancing the turnover of the oxidized NAD+ from NADH, OCNs would increase the volume of glycolytic energy metabolism and lactate generation in cellular models.

Also, considering the protective ability of these OCNs to improve the efficiency of electron transport to inhibited mitochondrial complexes and reduce damaging effects of superoxide radical, we assessed if OCNs could improve parameters of mitochondrial respiratory function basally and within the context of TBI complicated by contusion. Contusions are defined by hemorrhage that occurs when the vasculature is damaged in addition to injury to the brain parenchyma. In TBI with contusion, decreased oxygen supply and a toxic accumulation of excitatory neurotransmitters disrupt the mitochondrial membrane potential in neurons, glia, and endothelial cells, leading to increased ROS generation and the loss of ATP and NAD^+^ regenerative capacity.^[^ ^20^ ^]^ Elevated ROS levels contribute to oxidative damage to cellular components (i.e., DNA, protein, plasma membrane lipids), decrease the efficiency of mitochondrial energy metabolism, and mediate the loss of vascular tone autoregulation, immediately following trauma as well as persisting upon the restoration of blood flow during resuscitative efforts.^[^ ^17^, ^20^, ^21^, ^22^ ^]^ These injuries drive a concomitant increase in the activity of homeostatic pathways such as PARP‐mediated DNA damage repair and sirtuin‐mediated stress response pathways which further deplete intracellular NAD^+^ stores.^[^ ^23^, ^24^ ^]^ As such, interventions that decrease ROS levels intra‐ and extracellularly while also improving the efficiency of mitochondrial energy metabolism may support cellular recovery following TBI, thereby mitigating the damaging effects of TBI on brain tissue and vasculature.

In this report, we utilized extracellular flux analysis to characterize differences in oxygen consumption rates (OCR) and extracellular acidification rates (ECAR) in cultured murine brain endothelioma cells (bEnd.3) after treatment with OCNs, basally and following the addition of inhibitors of mitochondrial function and respiration.^[^ ^2^, ^25^, ^26^ ^]^ We selected this cell type to study given their critical role in the regulation of cerebral perfusion and maintaining the functional integrity of the blood‐brain barrier.^[^ ^27^, ^28^ ^]^ Our prior work established that our nanoparticles were able to rapidly restore cerebral perfusion impaired by TBI, reducing lesion size and improving functional outcomes in reperfusion injury models.^[^ ^17^, ^18^ ^]^ With the goal of extending the applications of OCNs to the toxic consequences of brain hemorrhage, we functionalized the activated charcoal‐derived OCN with the iron chelator deferoxamine for hemorrhage‐specific treatment through conjugation to available carboxyl groups, which are also utilized to covalently bond NH_2_‐PEG. Hemorrhage dramatically increases free iron levels, responsible for both oxidative injury via the Fenton reaction and initiating ferroptosis, an iron‐mediated program of cell death.^[^ ^4^, ^29^ ^]^ In this report, we investigated the metabolic activities of PEG‐cOACs and deferoxamine‐conjugated PEG‐cOACs (DEF‐PEG‐cOACs) in cell culture and in a rat model of moderate‐to‐severe traumatic brain injury complicated by hemorrhage, to assess the therapeutic translatability of these OCNs.

## 2 Results

### 2.1 Effects of OCNs on Cellular Oxygen Consumption Rate Dynamics

**Figure 1:**
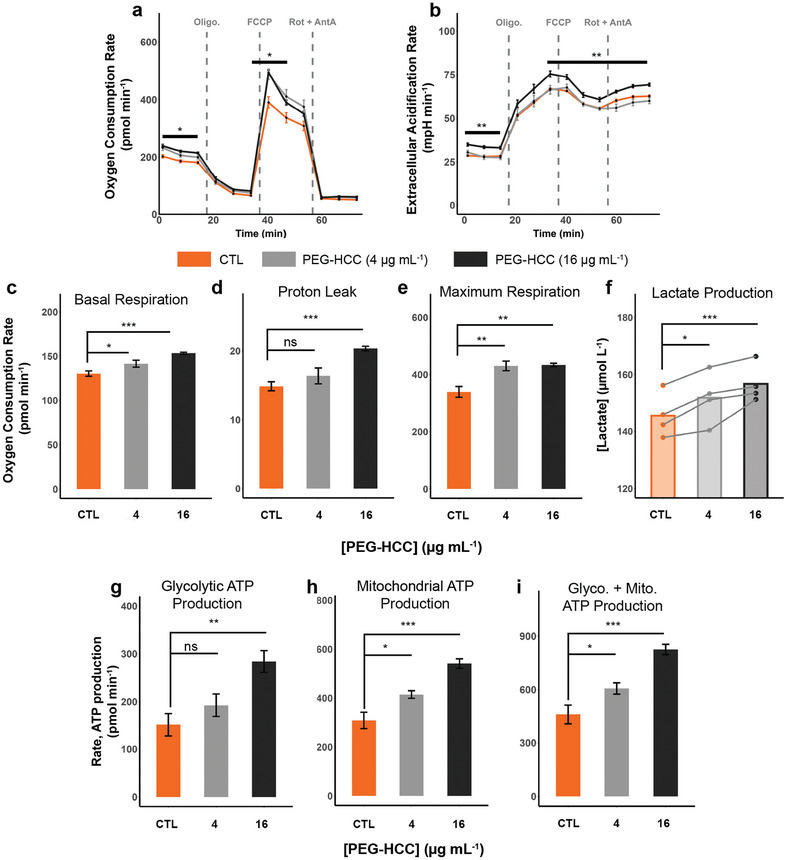
OCNs induce pro‐aerobic and pro‐glycolytic shifts in bEnd.3 cell energy metabolism. A) Oxygen consumption rate (OCR) and b) extracellular acidification rate (ECAR) are concurrently increased in bEnd.3 brain endothelial cells pretreated with PEGylated hydrophilic carbon clusters (PEG‐HCC) at therapeutically relevant dosages of 4 and 16 µg mL^−1^ versus untreated control (CTL). OCR is a measure of mitochondrial oxidative metabolism and ECAR is a measure of glycolytic rate. Higher OCR values indicate an aerobic shift in cellular energy metabolism, reflected in c) higher basal respiratory rate (difference between initial baseline and minimum OCRs following the addition of rotenone and antimycin A), d) higher proton leak (a protective adaptation against mitochondrial ROS generation, calculated as the difference between minimum OCR following oligomycin injection and OCR after the addition of rotenone/antimycin A) and e) maximal respiratory rate (difference between the maximal OCR following the addition of the uncoupling reagent FCCP and OCR after the addition of rotenone/antimycin A). Higher ECAR values indicate a glycolytic shift in energy metabolism, which is reflected in f) higher extracellular lactate levels versus CTL in 4 independent assays (represented by connected dots) following 24 h treatment of bEnd.3 cells with PEG‐HCCs. Specialized extracellular flux assays for the measurement of cellular ATP bioenergetics further reveal that PEG‐HCC treatment increased g) glycolytic ATP production, h) mitochondrial ATP production, and i) total (mitochondrial + glycolytic) cellular ATP production in a dose‐dependent manner. These findings suggest that OCNs induce pro‐aerobic and pro‐glycolytic shifts in cellular energy metabolism that persist through insults to mitochondrial function. Mean + SEM, one‐way ANOVA; *p < 0.05; **p < 0.01; ***p < 0.001.

To test the hypothesis that PEG‐HCC treatment improves parameters of mitochondrial respiratory function in bEnd.3 murine brain endothelial cells, we performed a mitochondrial stress assay using an Agilent Seahorse XFe96 Flux Analyzer. This assay measures oxygen consumption rate (OCR) and extracellular acidification rate (ECAR) at baseline and following the additions of 1 µm oligomycin (an inhibitor of ATP synthase), 1 µm carbonyl cyanide‐*p*‐trifluoromethoxyphenylhydrazone (FCCP, an uncoupling agent that dissipates the proton gradient across the inner mitochondrial membrane) and 0.75 µm rotenone and antimycin A (inhibitors of Complex I and III respectively). Within this general trend in OCR levels, bEnd.3 cells pre‐treated with 4 and 16 µg mL^−1^ PEG‐HCCs exhibited significantly elevated basal OCRs relative to control cells (CTL) treated with phosphate‐buffered saline (**Figure** **1**a; Figure S1, Supporting Information). Cells treated with PEG‐HCCs also showed a higher minimal OCR level after the addition of oligomycin and a higher maximal OCR upon the addition of FCCP (Figure 1a; Figure S1, Supporting Information). The finding that PEG‐HCC pre‐treatment increased OCR initially and immediately after oligomycin administration was reproduced across four independent mitochondrial stress assays at a treatment dose of 4 µg mL^−1^, while also showing that this treatment significantly increases mitochondrial proton leak (Figure S2A–C, Supporting Information). Proton leak is believed to be an adaptation to oxidative phosphorylation by which the uncoupling of the mitochondrial proton gradient can reduce the excess generation of ROS characteristic of impaired mitochondrial respiration.^[^ ^30^ ^]^ As such, this finding supports the hypothesis that PEG‐HCCs can function as a protective buffer against insults that negatively influence mitochondrial function, while also demonstrating the ability of PEG‐HCCs to promote an aerobic shift in cellular energy metabolism that persists through the inhibition of mitochondrial oxidative respiration.

### 2.2 OCNs Promote a Pro‐Glycolytic Shift in Energy Metabolism

We sought to investigate the relationship between PEG‐HCC pre‐treatment and glycolytic energy metabolism in bEnd.3 cells by analyzing ECAR values throughout the mitochondrial stress assay. ECAR is primarily driven by cellular lactic acid and CO_2_ generation, rises with the oligomycin‐induced inhibition of mitochondrial ATP generation, and is thus regarded as a robust measure of glycolytic metabolism.^[^ ^26^ ^]^ We observed that pre‐treating cells with 16 µg mL^−1^ PEG‐HCCs caused an assay‐wide elevation in ECAR values relative to the cells treated with 4 µg mL^−1^ PEG‐HCCs as well as the no‐treatment control (Figure 1b; Figure S3, Supporting Information). Furthermore, PEG‐HCC‐treated cells exhibited greater levels of lactate secretion into the extracellular medium across four independent assays (Figure 1f), increasing extracellular lactate levels by 6.3 and 11.1 µm on average with 4 and 16 µg mL^−1^ PEG‐HCC treatment respectively. This supports the finding that PEG‐HCC treatment induces a glycolytic metabolic shift in bEnd.3 cells consistent with an elevated ECAR profile. Together, these findings demonstrate that PEG‐HCCs can maintain cellular oxidative and glycolytic energy metabolism in response to external stresses, modeled here through oligomycin, rotenone, and antimycin A administration.

To better understand the effects of PEG‐HCC treatment on cellular energy metabolism, we compared bioenergetic parameters of cellular oxidative and glycolytic energetic metabolism using bEnd.3 cells were pre‐treated with PEG‐HCC versus cells treated with methylene blue (MB). MB is a phenothiazine dye with an established role as a mitochondrial electron carrier and has been shown to increase basal and maximal respiration in HT‐22 hippocampal neurons.^[^ ^2^, ^31^ ^]^ We demonstrate that MB pre‐treatment of bEnd.3 cells increases basal (Figure 1c; Figure S4A, Supporting Information) and maximal respiration (Figure 1e; Figure S4B, Supporting Information) relative to control group cells, albeit only at the lower dose (1 µm). Treatment of cells with 16 µg mL^−1^ PEG‐HCC increases basal respiration relative to the control group cells at a magnitude that is not significantly different from the 1 µm MB treatment condition (Figure S4A, Supporting Information). While the 4 µg mL^−1^ PEG‐HCC dose was not associated with a significant increase in basal respiration, both PEG‐HCC doses (as well as 1 µm MB) increased maximum respiration relative to the control group cells (Figure S4B, Supporting Information). In addition, a comparable elevation in proton leak relative to the control group cells was observed in both the 1 µm MB and the 16 µg mL^−1^ PEG‐HCC treatment groups (Figure 1d; Figure S4C, Supporting Information). These findings indicate that the ability of PEG‐HCC treatment to enhance mitochondrial oxidative respiration in bEnd.3 is comparable to the known actions of MB treatment, providing a useful benchmark.

In addition to mitochondrial respiration, the effects of PEG‐HCC treatment on glycolytic metabolism were compared with those of MB using a glycolytic rate assay, which measures proton efflux rate initially and following the addition of rotenone/antimycin A and 2‐deoxyglucose (an inhibitor of hexokinase and therefore, glycolysis).^[^ ^25^ ^]^ While the basal glycolytic rate was not significantly elevated by PEG‐HCC treatment relative to the control group cells (Figure S5A, Supporting Information), the compensatory increase in glycolytic rate following the inhibition of mitochondrial respiration with rotenone and antimycin A was significantly higher with 16 µg mL^−1^ PEG‐HCC treatment relative to the control group cells (Figure S5B, Supporting Information). This suggests that in addition to directly protecting mitochondrial energy metabolism, PEG‐HCC treatment may also increase glycolytic metabolism in response to impaired mitochondrial function, thereby improving cellular energetic resilience to external stressors that hinder normal energy metabolism.

### 2.3 OCNs Enhance Glycolytic and Mitochondrial ATP Production Capacity

To further characterize the specific effects of PEG‐HCC treatment on glycolytic and mitochondrial energy metabolism relative to MB, we performed a real‐time ATP production assay using the Seahorse XFe96 Flux Analyzer, an assay which measures OCR and ECAR at baseline and following the addition of 1.5 µm oligomycin and 0.5 µm rotenone and antimycin A under specific extracellular media conditions that allow for the determination of glycolytic and mitochondrial fractions of cellular ATP production rates.^[^ ^25^ ^]^ We observed that pre‐treatment of cells with 16 µg mL^−1^ PEG‐HCCs as well as with 1 and 10 µm MB significantly elevated the glycolytic ATP production rate relative to control group cells (Figure 1g; Figure S6A, Supporting Information). Additionally, a statistically significant dose‐dependent increase in glycolytic ATP production was observed across both the MB and PEG‐HCC treatment groups, with the higher dose condition exhibiting a higher glycolytic ATP production rate (Figure 1g; Figure S6A, Supporting Information). In comparison, both PEG‐HCC and MB‐treated cells (in both doses for each) exhibited a significant increase in mitochondrial ATP production relative to control group cells (Figure 1h; Figure S6B, Supporting Information). No significant difference in mitochondrial ATP production rate was observed between 1 and 10 µm MB treatment, whereas the higher PEG‐HCC dose was associated with a higher mitochondrial ATP production rate (Figure S6B, Supporting Information). This dose‐dependent increase in cellular ATP production is also observed in the total (glycolytic + mitochondrial) ATP production rates, in which the higher PEG‐HCC dose was associated with a higher total ATP production rate relative to the lower dose and the control group cells (Figure 1i) – a dose‐dependent effect not observed among the MB treatment groups (Figure S6C, Supporting Information). This is further supported by the decreased basal and maximal OCR observed in cells treated with 10 µm MB relative to 1 µm MB, which may indicate an oversaturation of oxidative respiration that is counterproductive to mitochondrial ATP generation (Figure S1, Supporting Information). As such, in addition to further supporting the role of PEG‐HCC treatment in enhancing glycolytic‐ and mitochondrial‐driven energy metabolism, these findings also suggest that PEG‐HCCs may have a greater capacity to increase mitochondrial ATP production than MB at higher doses unexplored here.

### 2.4 Synthesis and Characterization of DEF‐PEG‐cOACs

**Figure 2:**
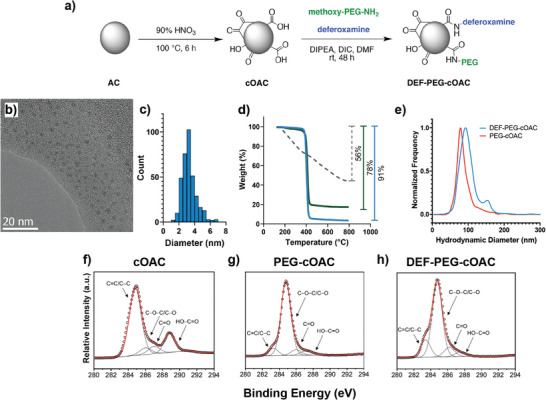
Synthesis and characterization of DEF‐PEG‐cOACs. a) Reaction scheme for the synthesis of DEF‐PEG‐cOACs from cOACs derived from fuming nitric acid oxidation of sieved cocoanut shell‐derived activated charcoal. b) Representative high‐resolution transmission electron microscopy of cOACs (scale bar 20 nm). (c) Size distribution of cOAC particles (mean: 3–3.5 nm; maximum: 7 nm; *n* = 360 particles). d) Thermogravimetric analysis of cOACs showed a mass loss of 56% (dashed line), PEG‐cOACs (blue line; 91%), and DEF‐PEG‐cOACs showing a mass loss of 78% (green line). e) Nanoparticle tracking analysis of PEG‐cOACs (mean: 89 nm; mode: 78 nm) and DEF‐PEG‐cOACs (mean: 94 nm; mode: 89 nm). f–h) High‐resolution X‐ray photoelectron spectroscopy C 1s spectrum of cOACs, PEG‐cOACs, and DEF‐PEG‐cOACs.

In a previous report by McHugh et al., we presented the synthesis of oxidized activated charcoal which gave excellent biocompatibility and potent antioxidant activity similar to PEG‐HCCs. We wished to see if these nanoparticles could lessen the harmful effects of traumatic brain injury and hemorrhage, an important driver of which is a toxic extracellular accumulation of iron that contributes to the generation of damaging reactive oxygen species and potentially the cell death pathway ferroptosis long after the initial injury.^[^ ^32^ ^]^ To this end, we prepared a deferoxamine‐functionalized derivative, DEF‐PEG‐cOAC, based on beneficial effects shown previously for DEF‐PEG‐HCCs reported by Dharmalingam et. al. in mitigating the toxic effects of a brain hemorrhage (24). Deferoxamine is a chelator that is FDA‐approved to treat diseases of iron overload,^[^ ^29^ ^]^ and our group has previously reported that deferoxamine‐functionalized oxidized PEG‐HCCs protected cells from iron‐dependent cytotoxic processes such as DNA damage and ferroptosis.^[^ ^14^ ^]^ As shown in **Figure** **2**a, coconut‐derived activated charcoal powder was sieved to collect a fraction with grains less than 20 µm in size and oxidized using fuming nitric acid at 140 °C to yield oxidized activated charcoal (cOAC).

Following the fuming nitric acid oxidation of 20 µm sieved coconut‐activated charcoal, high‐resolution TEM revealed nanoparticles with an average diameter of 3‐3.5 nm with a maximum diameter of 7 nm (Figure 2b,c). Simultaneous coupling of methoxy‐PEG‐NH_2_ and deferoxamine with EDC/NHS in MES buffer yields the DEF‐PEG‐cOAC nanoparticle. Thermogravimetric analysis of cOACs, a representative batch of PEG‐cOACs, and DEF‐PEG‐cOACs showed a loss of mass over a range of 100 C to 800 C (Figure 2d). the PEGylated nanoparticles underwent a rapid loss of mass at 400 °C due to the decomposition of PEG. We found that cOACs lost 56% of their mass whereas PEG‐cOACs and DEF‐PEG‐cOACs lost 91% and 78%, respectively. This is consistent with deferoxamine conjugated to the particle in place of PEG‐NH_2_ because deferoxamine has a lower molecular weight (561 Da) than 5000 Da PEG‐NH_2_ but competes with PEG‐NH_2_ for the same conjugation sites on the nanoparticle yielding an overall lower total weight. Nanoparticle tracking analysis of the DEF‐PEG‐cOACs (Figure 2e) gave an average particle diameter of 100 nm and a mode of 89 nm. A comparison of the deconvolved high‐resolution X‐ray photoelectron (XPS) C 1s spectra of cOACs (Figure 2f), PEG‐cOACs (Figure 2g), and DEF‐PEG‐cOACs (Figure 2h) gave rise to distinct spectra (analysis shown in **Table** **1**). XPS survey spectra of the C, N, and O peaks showed a total N atom percentage of 1.7% for the DEF‐PEG‐cOACs, and 0.6% for PEG‐cOACs, compared to cOACs with 0%.

**Table 1:** Elemental composition and oxygen‐functionality distribution of cOACs and DEF‐PEG‐cOACs calculated from XPS survey and high‐resolution C 1s peak measurements.

	Atom percentage [at%]		
Functional Group	cOAC [at%]	PEG‐cOACs [at%]	DEF‐PEG‐cOAC [at%]
C═C/C─C	66.2	7.9	16.3
C─O─C/C─O	12.1	79.9	70.0
C═O	6.6	6.5	9.4
O─C═O	15.2	5.8	4.2
N	0	0.6	1.7
C:O Ratio	2.6	1.9	2.1

Deferoxamine has 6 N atoms per molecule whereas PEG‐NH_2_ only has the terminal ─NH_2_, therefore, deferoxamine‐functionalized nanoparticles should have more N content than the PEG‐only nanoparticles, indeed here we see the result of this in Table 1. Likewise, we expect the C:O ratio of a PEGylated particle to be lower than the cOAC particle because, for approximately every two atoms of C, there is one atom of O in the PEG chain. Because most of the cOAC is composed of carbon, the addition of PEG will decrease the C:O ratio. DEF‐PEG‐cOAC particles have a higher C:O ratio most likely because the smaller molecules of deferoxamine substitute for some of the PEG‐NH_2_, leading to a higher C:O ratio instead.

**Figure 3:**
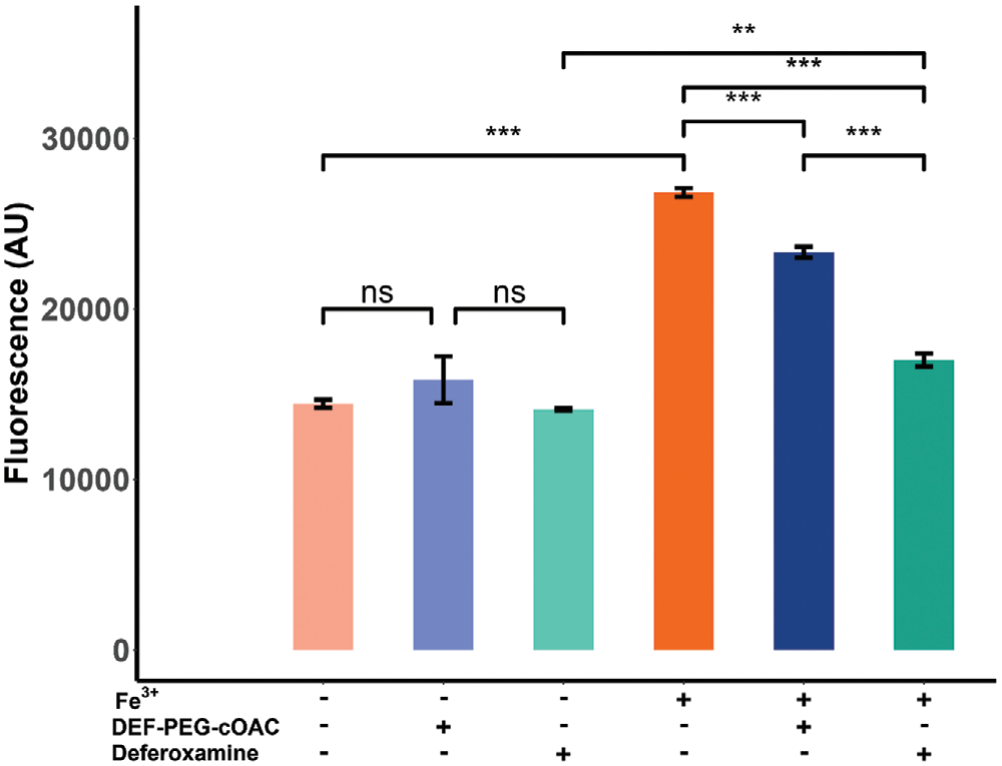
Deferoxamine‐conjugated oxidized carbon nanozymes (DEF‐PEG‐cOACs) demonstrate iron chelation effect in bEnd.3 cells. Mouse brain endothelial (bEnd.3) cells were cultured in media with and without iron (III) chloride (50 µm), DEF‐PEG‐cOACs (4 µg mL^−1^), and the iron chelator deferoxamine (50 µm). Free deferoxamine was estimated as 100X higher molarity than when particle bound. Intracellular free iron was measured using fluorescence microscopy with FerroOrange, a fluorescent probe sensitive to unbound Fe^2+^. Treatment of iron cells with DEF‐PEG‐cOACs and deferoxamine in the presence of iron (III) chloride significantly decreases FerroOrange fluorescence, reflecting decreased intracellular free iron. DEF‐PEG‐cOACs are ≈63‐fold more potent per mole of deferoxamine than free deferoxamine. No significant difference in fluorescence between DEF‐PEG‐cOAC and deferoxamine‐treated cells was observed in the absence of media iron supplementation, suggesting that DEF‐PEG‐cOACs and deferoxamine do not significantly interfere with FerroOrange fluorescence. *N* = 3 technical replicates. One‐way ANOVA. *p < 0.05; **p < 0.01; ***p < 0.001; “ns” indicates a non‐significant comparison.

To assess the ability of deferoxamine‐conjugated, PEGylated oxidized carbon nanozymes (DEF‐PEG‐cOACs) to chelate excess extracellular iron, we measured intracellular free (unbound) iron levels in bEnd.3 cells that were cultured in media supplemented with 50 µm iron (III) chloride, DEF‐PEG‐cOACs (4 µg mL^−1^), and deferoxamine (50 µm). Deferoxamine is a chelator with a high affinity for ferric (Fe^3+^) iron and thus prevents its reduction to the more toxic ferrous (Fe^2+^) form^[^ ^33^ ^]^ – for this reason, and because most unbound extracellular iron exists in the ferric state at physiological pH,^[^ ^34^ ^]^ iron (III) chloride supplementation was chosen to mimic iron overload. Using fluorescence microscopy with FerroOrange, a fluorescent probe specific to unbound Fe^2+^, intracellular free iron levels were quantified using feature‐based image analysis.^[^ ^35^ ^]^ In bEnd.3 cells cultured in iron‐supplemented media, treatment with DEF‐PEG‐cOACs and deferoxamine significantly decreased intracellular unbound Fe^2+^ levels relative to cells not treated with either effector (**Figure** **3**; Figure S8, Supporting Information). Also, no significant difference in fluorescence signal intensity between DEF‐PEG‐cOAC‐treated, deferoxamine‐treated, and untreated bEnd.3 cells not cultured in iron supplement media were observed, possibly due to most intracellular Fe^2+^ being bound to ferritin under normal physiological conditions and thus decreasing the availability of free iron to be chelated.^[^ ^32^, ^33^, ^34^ ^]^ This lack of difference also suggests that neither DEF‐PEG‐cOAC nor deferoxamine at their administered concentrations quench or independently activate FerroOrange fluorescence, thus supporting the validity of this fluorescence‐based method of free iron detection. However, an important caveat of this method is that the thresholding method (Otsu) for cell region identification may fail to detect weakly fluorescent cells and thus may underestimate the decrease in fluorescence due to chelation. This motivates future investigations into the iron chelation ability of DEF‐PEG‐cOAC using more robust quantitative methods such as mass spectrometry.

With a delivered concentration of 4 µg mL^−1^ DEF‐PEG‐cOAC, the amount of deferoxamine conjugated to cOACs in solution was calculated using the method described by Dharmalingam et al. to be the equivalent of 0.2 µm. We observed a 20% reduction in fluorescence compared to the Fe^3+^‐treated control but observed an 80% reduction compared to the 50 µm deferoxamine. The primary implication of this finding is that a 100× lower dose of deferoxamine‐conjugated has 63× the effect of deferoxamine alone. There are caveats to this, however: the concentration of intracellular iron is not known, and it is also not known so the effect of deferoxamine may be underestimated in the 50 µm condition. However, because the 50 µm Fe^3+^ treatment with 50 µm deferoxamine does not return the fluorescence signal to baseline it is reasonable that not all the deferoxamine was available to chelate the excess iron. A quantitative study of iron uptake by DEF‐PEG‐cOACs is necessary in order to resolve these unknowns.

### 2.5 Comparison of Pro‐Aerobic and Pro‐Glycolytic Effects of OCNs

**Figure 4:**
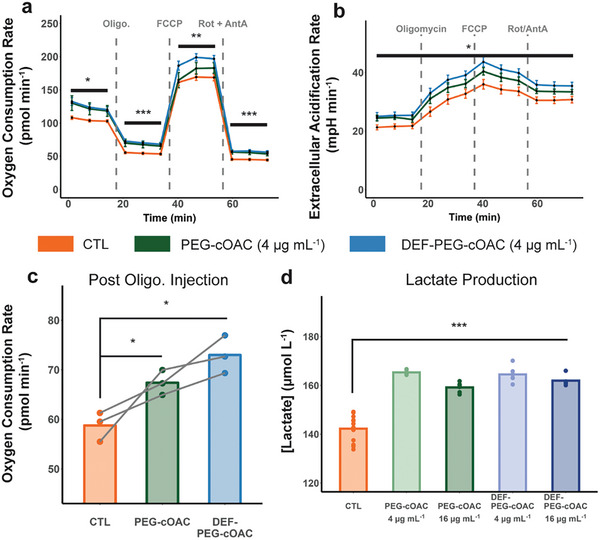
Pro‐energetic effects of deferoxamine‐functionalized OCNs a) Synthesis schematic for PEGylated oxidized carbon nanozymes synthesized from coconut‐derived activated charcoal (PEG‐cOACs) and PEG‐cOACs functionalized with the iron chelator deferoxamine (DEF‐PEG‐cOACs). PEG‐cOACs and DEF‐PEG‐cOACs increase bEnd.3 cell b) oxygen consumption rates (OCR) and c) extracellular acidification rates (ECAR), indicative of pro‐aerobic and pro‐glycolytic shifts in energy metabolism respectively. Of note, the increase in OCR versus untreated (CTL) cells is greater with DEF‐PEG‐cOAC treatment, suggesting an improvement upon the pro‐aerobic shift facilitated by PEG‐cOACs. d) Higher OCR in PEG‐ and DEF‐PEG‐cOAC‐treated bEnd.3 cells after the addition of the ATP synthase inhibitor oligomycin (2 µm) suggests that the pro‐aerobic effects of PEG‐ and DEF‐PEG‐cOACs persist through negative stressors of mitochondrial respiration. e) Higher extracellular lactate release as a result of 24 h PEG‐cOAC and DEF‐PEG‐cOAC treatment of bEnd.3 cells support a pro‐glycolytic role for these OCNs at a comparable magnitude to that of PEG‐HCCs. Mean + SEM, one‐way ANOVA; *p < 0.05; **p < 0.01; ***p < 0.001.

Next, we assessed the metabolic effects of cOACs modified with PEG and deferoxamine conjugates. We observed that bEnd.3 cells pretreated with PEG‐cOACs and deferoxamine‐conjugated PEG‐cOACs (DEF‐PEG‐cOACs) caused higher basal oxygen consumption rates (**Figure** **4**a) and ECAR throughout the mitochondrial stress assay (Figure 4b), similar to what was seen with PEG‐HCC pretreatment. Furthermore, PEG‐ and DEF‐PEG‐cOAC pretreatment increased OCR following the addition of oligomycin relative to the untreated control in three independent experiments (Figure 4c) as well as higher levels of extracellular lactate release (Figure 4d). With regards to the latter, 4 µg mL^−1^ PEG‐ and DEF‐PEG‐cOAC treatment of cells increased extracellular lactate levels by 23.1 and 22.2 µm respectively (quantitation based on in‐vitro calibration curve using exogenously applied lactate; Figure S7, Supporting Information), suggesting an increase over the lactate increase observed with PEG‐HCCs.

### 2.6 Protection and Pro‐Energetic Effects of OCNs In a Model of Brain Trauma

**Figure 5:**
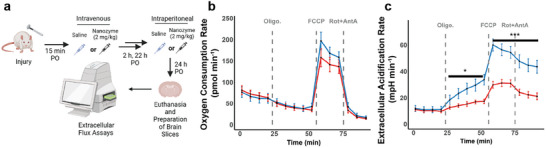
Effect of DEF‐PEG‐cOACs on mitochondrial respiration following traumatic brain injury. a) Schematic of rat model of in vivo contusion TBI. Cortical punch biopsy sections from rats subject to injury and treated with DEF‐PEG‐cOACs (2 mg kg^−1^, intravenously 15 min and intraperitoneally 2‐ and 22 h post‐injury) trended toward b) higher maximal oxygen consumption rates (OCR) and c) significantly higher extracellular acidification rates (ECAR) versus saline vehicle control (CTL, Veh.), indicating a concurrent aerobic and glycolytic shift in energy metabolism that may signify resilience to the effects of external trauma on cellular energy metabolism. Mean + SEM, one‐way ANOVA; * *p <* 0.05; ** *p <* 0.01; *** *p <* 0.001. Figure using Biorender.com.

Having demonstrated the efficacy of DEF‐PEG‐cOACs in enhancing aerobic and glycolytic energy metabolism in endothelial cells, we wished to investigate if this treatment would confer a similar resilience to derangements in cellular energy metabolism in a rat model of traumatic brain injury. Following mechanical trauma resulting in hemorrhagic contusion, rats were administered either OCN (2 mg kg^−1^) or an equivalent volume of saline intravenously (15 min post‐injury) and intraperitoneally (2‐ and 22 h post‐injury; **Figure** **5**a). Cortical punch biopsy sections from rats subjected to mechanical brain trauma were analyzed using extracellular flux analysis as previously described.^[^ ^36^, ^37^, ^38^ ^]^ Sections of brain tissue from rats that were administered DEF‐PEG‐cOACs prior to injury exhibited a trend toward higher maximal OCR (Figure 5b) and significantly higher ECAR (Figure 5c) relative to rats treated with the saline vehicle.

**Figure 6:**
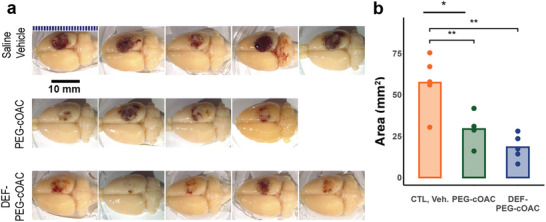
Protective effects of deferoxamine‐functionalized OCNs in a rat model of contusion‐based hemorrhagic traumatic brain injury (TBI) a) Parietal lobe hemorrhagic lesions in PEG‐ and DEF‐PEG‐cOAC‐treated rats post‐cortical injury exhibited significantly reduced b) area (mm^2^) relative to saline vehicle‐treated animals, suggesting that PEG‐ and DEF‐PEG‐cOAC treatment confers protection and reduces the deleterious effects of contusion TBI, with a non‐significant trend toward increased efficacy with DEF‐PEG‐cOAC treatment (48.7% vs 68.2% PEG‐cOAC vs DEF‐cOAC‐PEG respective reduction in hematoma area; NS difference). *N* = 4–5 animals/group. One‐way ANOVA. * *p <* 0.05; ** *p <* 0.01; *** *p <* 0.001.

The area of the parietal lobe hemorrhagic lesion site in rats treated with DEF‐PEG‐cOACs was 68.2% lower than that of vehicle control while PEG‐cOAC treatment resulted in 48.7% reduction (NS difference between OCNs, both significant versus saline treatment) (**Figure** **6**a,b). The reduction of lesion area trended toward greater with DEF‐PEG‐cOAC treatment as compared with PEG‐cOAC, however, additional experiments will be necessary to establish the additive advantage in this outcome measure due to DEF‐conjugation. An individual brain randomly obtained from each treatment group was subjected to histological analysis (Supplementary Experimental Section and Figure S9, Supporting Information). While considerable tissue distortion and injury are seen with TBI and saline treatment, both OCNs reduced apparent tissue distortion with some trend toward a smaller cortical defect with the DEF‐PEG‐cOAC OCN, although a systematic analysis is required to substantiate any differential effect. However, overall, the substantially lower manifestation of physical injury suggests that OCN treatment confers protection to brain and vascular tissue in this hemorrhagic contusion model of traumatic brain injury. As compensatory glycolytic energymetabolism is an important mechanism of cellular resilience in the context of traumatic brain injury,^[^ ^1^, ^39^ ^]^ an OCN‐mediated increase in glycolytic flux may represent a mechanism of cell and tissue protection, motivating targeted future investigations.

## 3 Discussion

In this report, we demonstrate that OCNs (PEG‐HCCs, PEG‐cOACs, DEF‐PEG‐cOACs) increase parameters of oxidative and glycolytic energy metabolism. Our finding that PEG‐HCC‐treated bEnd.3 cells maintain higher OCRs following the addition of oligomycin supports our earlier characterization of PEG‐HCCs as mitochondrially‐protective nanozymes.^[^ ^4^ ^]^ The enhanced OCR observed in PEG‐HCC‐treated cells, basally and in the presence of oligomycin shows a parallel to that of MB, an electron carrier that participates in mechanisms of alternative mitochondrial electron transfer that have been demonstrated to rescue acute impairments of mitochondrial function.^[^ ^2^, ^40^ ^]^ Studies from other groups have emphasized the ability of MB treatment to rescue complex I and complex III inhibition^[^ ^41^ ^]^ by oxidizing NADH to NAD+ with MB to form leuko‐MB, and electron cycling with electron acceptors such as oxygen.^[^ ^2^, ^40^ ^]^ A recent study by Bouillaud et. al. has indicated that MB elevates cellular oxygen consumption even with the oligomycin‐induced abrogation of mitochondrial oxidative phosphorylation,^[^ ^42^ ^]^ a finding seen in our report with OCNs as well. Oligomycin directly inhibits proton passage through the inner mitochondrial membrane due to its binding to the F0 subunit of ATP synthase, thus inhibiting mitochondrial respiration in the absence of uncoupler proteins that facilitate transmembrane proton leak.^[^ ^30^, ^43^ ^]^ As such, the concomitant enhancement seen in transmembrane proton leak with PEG‐HCC treatment may represent an increase in the efficiency of mitochondrial inner membrane electron transfer reactions by providing an ATP synthase‐independent outlet for proton re‐entry into the matrix.^[^ ^30^ ^]^ This finding, together with the ability of PEG‐HCCs to reduce cytochrome c and thereby rescue cyanide‐induced complex IV inhibition (as previously characterized) may thus provide an explanation for the enhancement of oxygen consumption rate and mitochondrial ATP production.

**Figure 7:**
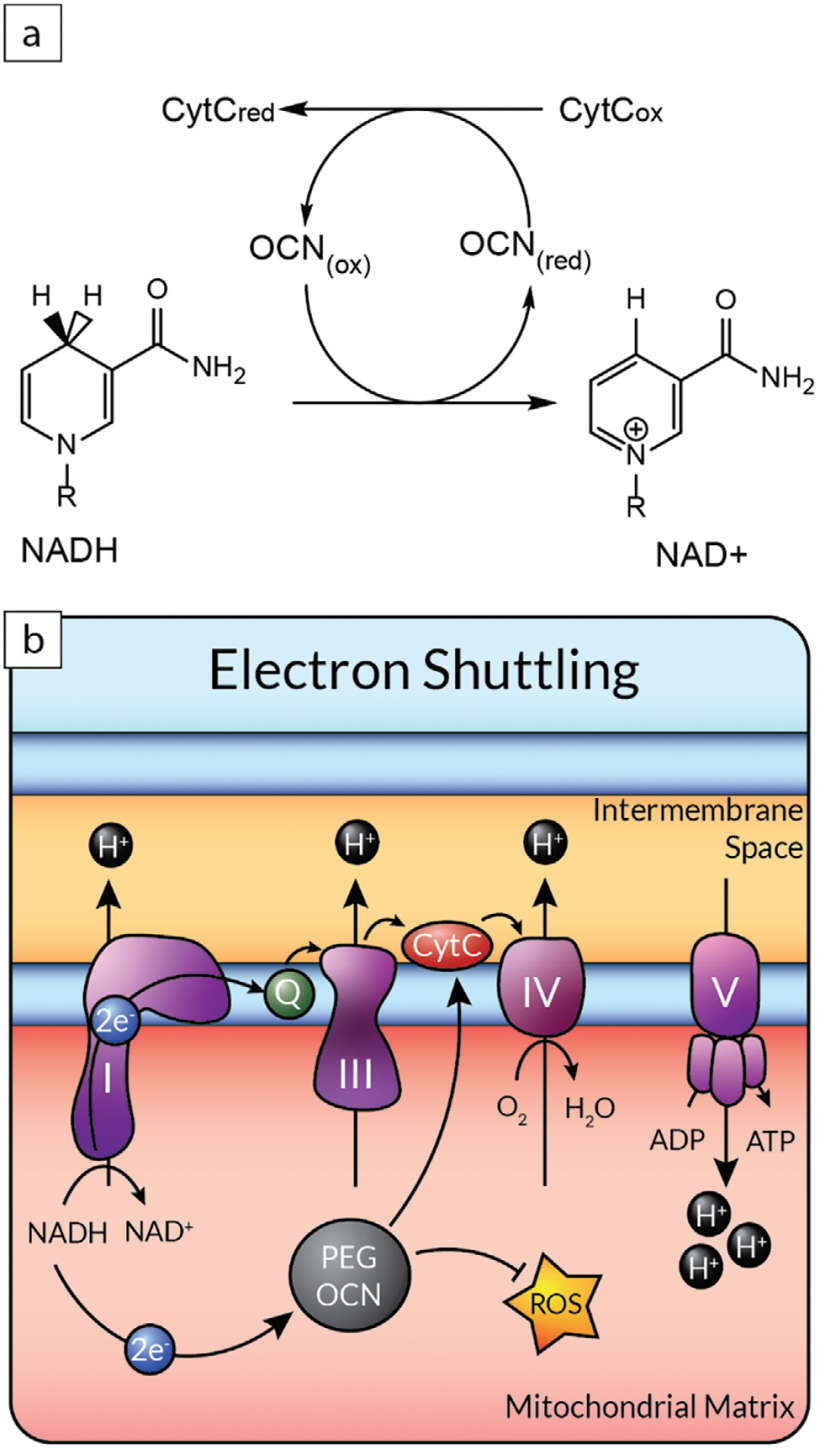
Proposed mechanism of OCN‐catalyzed electron transfers within mitochondrial respiration. a) OCNs oxidize the reduced electron carrier NADH to NAD+ and facilitate the transfer of electrons to oxidized cytochrome c (CytC_ox_). Reduced cytochrome c (CytC_red_) is an important mitochondrial electron carrier protein that transfers electrons between coenzyme Q‐cytochrome c reductase (mito. respiratory complex III) and cytochrome c oxidase (mito. respiratory complex IV), thereby promoting the formation of a proton gradient between the mitochondrial intermembrane space and the matrix. b) OCN‐catalyzed electron transfer between NADH and CytC may bypass inhibited respiratory complexes, thus preserving the intermembrane proton gradient necessary for ATP generation. Electron transfer to OCNs may also enable OCN‐mediated dismutation of mitochondrially‐toxic reactive oxygen species (ROS).

The precise mechanism of how OCNs oxidize NADH to NAD+ is not currently known ‐ however, in previous reports, we found spectroscopically that NADH is consumed by PEG‐HCCs in the presence of cytochrome C or the cell viability dye resazurin. Electron paramagnetic resonance spectrometry also revealed an increase in the PEG‐HCC radical singlet electron parametric resonance signal in the presence of NADH. We expect that the OCNs undergo a cyclic reaction between the electron donor (NADH) and electron acceptor (Cytochrome C) (**Figure** **7**a). We cannot determine if there is a radical step involved in the reaction – however, prior work showed a 2:1 ratio of cytochrome C reduced to NADH oxidized. Moreover, as we reported previously, this action may be responsible for the cytoprotective effects against cyanide poisoning.

However, there exist important differences between MB and OCNs in their ability to promote aerobic energy metabolism (Figure 7b). In this report, we demonstrate that bEnd.3 cells treated with 10 µm MB exhibit significantly reduced basal and maximal respiratory rates as well as decreased proton leak as compared to cells treated with 1 µm MB. This suggests a limited mitochondrial tolerance for MB and potentially even toxic effects that decrease the efficiency of oxidative phosphorylation at higher MB doses, which is consistent with our previously reported finding that bEnd.3 cells exhibit decreased viability when co‐cultured with MB at doses higher than 5 µm.^[^ ^4^ ^]^In contrast, this decrease in basal and maximal respiratory rates with higher effector dosage is not observed with OCNs, with higher OCN dosage even improving mitochondrial ATP generation relative to the lower dose. These differences in trends with effector dosages suggest that OCNs mediate distinct effects on mitochondrial energy metabolism from those of MB and that there may exist a greater physiological tolerance to OCNs than MB at higher doses. As such, future experiments are needed to establish optimal dose ranges for OCNs in different cell types with regard to improvements in mitochondrial energy metabolism and the protection of cellular (and mitochondrial) viability.

The present findings of increased glycolytic flux and glycolytic ATP production in PEG‐HCC‐treated cells extend this protective ability to cytosolic energy metabolism as well, enhancing glycolytic and aerobic energy metabolism basally and in the presence of stressors to mitochondrial function. The promotion of a glycolytic shift simultaneously with enhanced aerobic respiratory capacity is consistent with a paradigm of aerobic glycolysis, an energetic state that is driven by an intracellular drive to regenerate NAD^+^ from NADH.^[^ ^44^ ^]^ NAD^+^ is a critical coenzyme that drives substrate‐level and oxidative phosphorylation through its essential roles in glycolysis, fatty acid oxidation, and the Krebs cycle.^[^ ^45^ ^]^ When the mitochondrial capacity to oxidize NADH to NAD^+^ is limited, such as in nutrient‐limiting conditions following injury to tissues,^[^ ^46^ ^]^ the fermentation of glycolytically‐derived pyruvate to lactate becomes the dominant intracellular pathway by which cellular NAD+ levels are replenished. However, the low yield of this process – 2 molecules of pyruvate (and subsequently, lactate) per molecule of glucose – as well as the dependence of glycolytic flux on the availability of NAD^+^ itself suggests that this compensatory mechanism of regenerating NAD^+^ may become quickly overwhelmed.^[^ ^47^, ^48^ ^]^ Within this context, the catalytic ability of OCNs to regenerate NAD^+^ from NADH in a glucose‐independent manner may provide a means by which glycolytic flux is enhanced, thus promoting cellular NAD+ regeneration and ATP production even in the presence of mitochondrial stressors.

This potential pro‐glycolytic mechanism has also been attributed to MB^[^ ^42^ ^]^ and is supported by enhanced glucose uptake with MB treatment in primary astrocytes^[^ ^40^ ^]^ and in HT‐22 mouse hippocampal cells^[^ ^40^, ^49^ ^]^ and in HT‐22 mouse hippocampal cells.^[^ ^49^ ^]^ Yet, it is likely that the pro‐glycolytic effects of OCNs arise from a different metabolic context than that of MB. The higher glycolytic flux and ATP generation observed in cells treated with 10 µm MB relative to 1 µm occur in concert with decreased basal (aerobic) respiration, suggesting that mitochondrial toxicity at higher MB doses could also explain its pro‐glycolytic effects. Also curiously, acute MB injection has been associated with a depression in ECAR values in HT‐22 cells.^[^ ^2^, ^31^ ^]^ While this would seem to contradict the findings presented in this report, it is important to note that the timescale of treatment is likely an important mediator of the pro‐glycolytic effects of MB and OCNs. The pro‐glycolytic shift seen in our report follows from a 24 h pretreatment of cells with effectors prior to extracellular flux analysis, and it is likely that metabolic adaptations dependent on the intracellular [NAD^+^]/[NADH] ratio occurred during this pretreatment interval. Such adaptations include NAD^+^‐dependent activation of SIRT5, which increases glycolytic flux through GAPDH demalonylation.^[^ ^50^, ^51^ ^]^ The contributions of anaplerotic reactions to Krebs cycle flux and the resultant increase in ECAR via carbon dioxide generation must also be considered, due to the wide range of metabolic reactions that depend on the [NAD^+^]/[NADH] ratio. This motivates further investigation into the potential modulation of other metabolic pathways by OCNs in future reports.

In concert with a glycolytic shift in energy metabolism, we report that bEnd.3 cells treated with OCNs exhibit greater levels of lactate release. This is consistent with our hypothesis that by facilitating NADH oxidation, OCNs increase the rate of lactate generation, a reaction that is coupled with glycolysis.^[^ ^52^ ^]^ Contrary to its perception as a “waste product” of anaerobic fermentation, lactate is a beneficial metabolite that supports cell and tissue energy metabolism under a wide range of environmental conditions.^[^ ^1^, ^20^, ^39^, ^52^ ^]^ Lactate generated by metabolically active cells is an energy‐rich substrate that cells can use to generate the pyruvate and NAD+ required to support mitochondrial and glycolytic energy metabolism. As such, lactate metabolism improves cellular energetic flexibility, defined as the ability of cells to efficiently harness multiple sources of energy in response to environmental or pathological stressors.^[^ ^53^ ^]^ Our choice of a bEnd.3 cellular model is relevant in this regard, as endothelial cells occupy an important niche as a regulator of tissue homeostasis in basal and pathophysiological states by way of their lactate‐releasing capacity. Endothelial cell‐derived lactate (alongside astrocytes) is a crucial metabolite that supports neuronal energy metabolism in the brain, and stroke‐induced upregulation of the lactate transporter MCT4 enhances extracellular lactate export.^[^ ^39^ ^]^ Endothelial cell upregulation of the lactate transporters MCT1 and MCT2 has also been shown to correlate with resilience to ischemia‐induced injury in the brain.^[^ ^54^ ^]^ In addition to supporting energy metabolism, a growing body of evidence suggests a role for lactate as an activator of important homeostatic mechanisms under injury conditions. These include the direct activation of the unfolded protein response and the Nrf2/Keap1 axis in an SH‐SY5Y neuroblastoma cell model, mechanisms that increase cellular resilience to oxidative stress, and dysregulated proteinogenesis seen in various models of neurotrauma.^[^ ^55^ ^]^ The role of endothelial cells as a “lactate reservoir” that balances the energetic demands of surrounding tissue and promotes tissue regeneration and angiogenesis^[^ ^56^ ^]^ holds promise as a therapeutic target for OCNs with beneficial implications for tissue homeostasis.^[^ ^53^ ^]^

**Figure 8:**
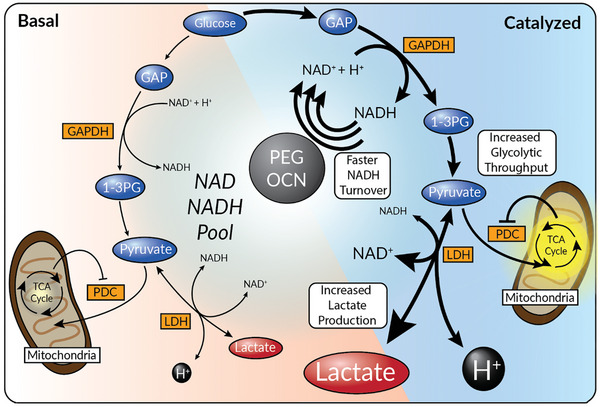
PEG‐OCNs increase the rate of lactate production by altering cytosolic metabolism. NADH metabolism in glycolysis is facilitated by glyceraldehyde‐3‐phosphate dehydrogenase (GAPDH) and lactate dehydrogenase (LDH). GAPDH produces NADH from the oxidation of glyceraldehyde‐3‐phosphate to 1,3‐phosphoglycerate, and LDH reversibly reduces pyruvate to lactate with NADH, and vice versa. The flux of pyruvate into the mitochondria is regulated by the tricarboxylic acid cycle which inhibits the pyruvate dehydrogenase complex when TCA intermediates increase beyond a threshold. In this report, we observed a large increase in extracellular acidification with a small change in mitochondrial oxygen consumption rate (OCR) in cells treated with PEG‐OCNs. The increase in extracellular acidification of PEG‐OCN‐treated cells can be in part accounted for by an increase in glycolytic rate caused by PEG‐OCNs oxidizing the NADH to NAD+ produced by GAPDH and increasing the rate of intermediate flux by increasing the concentration of substrate (NAD^+^) for GAPDH. The tricarboxylic acid cycle (TCA) rate limits mitochondrial pyruvate flux which leads to an accumulation of lactate and consequent H^+^ release because of pyruvate availability for LDH and a small increase in (OCR). The relative differences in intermediate flux and production are shown as changes in line width and font size, respectively.

Based on our understanding of how OCNs oxidize NADH to NAD+, we developed a hypothesis that can explain the elevated lactate levels. As shown in **Figure** **8**, OCNs increase glycolytic throughput by oxidizing NADH that was produced by GAPDH. GAPDH serves two purposes, it both oxidizes and phosphorylates glyceraldehyde‐3‐phosphate (GAP) to 1,3‐bisphosphoglycerate (1,3‐BPG). Because enzyme kinetics are dependent on the relative concentration of products to substrates, artificially decreasing the concentration of the product will increase the rate as the substrate is consumed. Therefore, changes to the [NAD^+^]/[NADH] ratio will increase the rate that GAPDH phosphorylates glyceraldehyde‐3‐phosphate (GAP) to 1,3‐bisphosphoglycerate (1,3‐BPG) insofar as inorganic phosphate and GAP are available. Consequently, this likely increases the molar concentration of glycolytic intermediates in the cell which eventually saturate the mitochondria and are finally stored as lactate.

**Figure 9:**
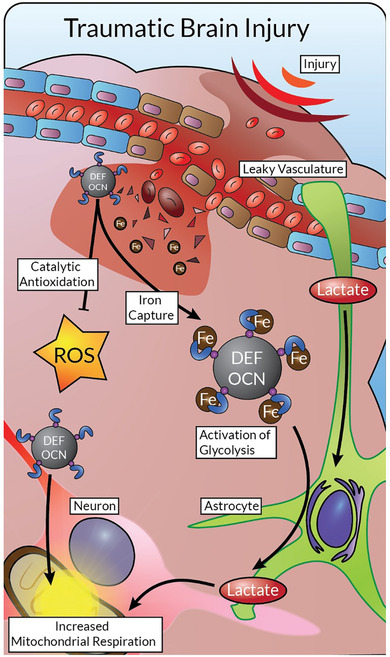
Proposed schematic of pleiotropic OCN‐mediated protection during hemorrhagic traumatic brain injury (TBI). Disruption of cerebral vasculature as a result of injury results in extravascular leakage and pooling of blood. Iron released from the breakdown of hemoglobin in blood generates cytotoxic reactive oxygen species (ROS) via the Fenton reaction, exacerbating cellular injury and decreasing the efficiency of neuronal energy metabolism. Deferoxamine‐functionalized OCNs may mediate cell and tissue protection during hemorrhagic TBI by i): chelating free iron, thereby limiting ROS generation, ii): catalyzing the dismutation of ROS, iii): rescuing injury‐inhibited aerobic (mitochondrial) respiration, and iv): stimulating glycolytic energy metabolism in neurons, astrocytes and endothelial cells, thus ensuring the generation of lactate that is transported to neurons via the endothelial/astrocyte‐neuron lactate shunt and utilized as an important adjunct energy source for mitochondrial ATP and NAD+ generation.

Furthermore, we report a significant decrease in both the area and intensity of cerebral hemorrhagic lesions following OCN treatment in a rat contusion model of hemorrhagic TBI. Contusions are a damaging consequence of TBI in which vascular disruptions due to mechanical forces cause the pooling of blood in the interstitium, increasing pressure on the surrounding tissue in the process.^[^ ^57^ ^]^ In addition to this physical mechanism of morbidity, pooled blood in hematomas is replete with toxic metabolites that contribute to the enhancement and propagation of tissue damage. As shown in **Figure** **9**, this includes excess iron that is released as a result of hemoglobin breakdown, which reacts with endogenous H_2_O_2_ to generate damaging hydroxyl radicals via the Fenton reaction.^[^ ^29^ ^]^

Both extra‐ and intracellular ROS generated from Fenton reactions contribute to the pathophysiology of traumatic brain injury (TBI) with hemorrhage.^[^ ^58^ ^]^ Extracellular hemin also activates microglia and promotes neutrophil infiltration into affected brain tissue, which increases the extracellular ROS burden via heightened NADPH oxidase‐mediated generation of superoxide and myeloperoxidase‐mediated generation of hydrogen peroxide.^[^ ^59^ ^]^ Increased ROS directly damages the network of endothelial tight junctions and surrounding extracellular matrix, while a reduction in energy harvesting capacity limits the scope of cellular recovery in the context of hemorrhagic TBI and initiates a cascade of cellular signaling events that culminate in apoptosis, ferroptosis, or necrotic cell death.^[^ ^60^ ^]^ This loss of endothelial cell integrity weakens the blood‐brain barrier, which facilitates the extravasation of blood and further exacerbates hemorrhage following TBI.

It is important to note that extracellular ROS generation and accumulation of toxic chemical species potentiate intracellular ROS generation and cellular injury. As a result of ROS‐mediated damage to the plasma membrane, necrotic cells release calcium ions and glutamate which directly compromise mitochondrial electron transport chain function and trigger the release of superoxide from inner membrane complexes I and III.^[^ ^1^, ^61^, ^62^ ^]^ Mitochondria are the dominant intracellular source of ROS, and the resulting “ROS burst” can overwhelm superoxide dismutases and aggravate lipid peroxidation and DNA damage, leading to cell death.^[^ ^63^ ^]^ Furthermore, the excess hydrogen peroxide produced as a result of superoxide dismutation can react with the simultaneous excess ferrous iron as a result of hemorrhage, resulting in a Fenton reaction as well as likely ferroptosis.^[^ ^29^ ^]^ As the origin and damaging effects of extra‐ and intracellular ROS generation within the context of hemorrhage‐associated TBI are intertwined, both sources must be considered together when proposing modalities to alleviate ROS‐mediated oxidative stress. We have previously reported on our OCNs’ ability to efficiently dismutate superoxide anion radicals in vitro and in in vivo models of reperfusion injury following reversible middle cerebral artery occlusion.^[^ ^4^, ^16^, ^19^, ^64^ ^]^ By virtue of their hydrophilic properties as well as their avid cellular uptake, OCNs may mitigate oxidative stress outside and inside the cell, respectively, thereby mitigating the extent of ROS‐mediated damage at a cellular and tissue‐wide level.^[^ ^18^, ^19^ ^]^

Addressing the distinct mechanisms – ROS toxicity and metabolic dysfunction – that drive the pathophysiology of hemorrhagic TBI necessitates a multi‐mechanistic approach.^[^ ^65^, ^66^ ^]^ The iron chelator deferoxamine mesylate (DEF) has demonstrated reduced neuronal death in a piglet model of intracranial hemorrhage.^[^ ^65^ ^]^ While results were mixed, DEF showed some promise as an intervention that improved modified Rankin Scale scores (a measure of neurological disability) relative to placebo following intracerebral hemorrhage in human participants of the i‐DEF clinical trial.^[^ ^66^ ^]^ However, the effects of DEF administration became apparent only after 90 days of treatment, and the doses employed in the animal models were toxic in the initial DEF human trials, leading to the lower doses employed in i‐DEF. The conjugation of DEF with OCNs offers a synergistic approach to address the iron‐induced toxicity and metabolic derangements observed in hemorrhagic TBI and because of avid cellular uptake, requires an estimated 100‐fold lower DEF dose than if used alone.^[^ ^14^ ^]^ In addition to their ability to chelate iron and quench ROS such as the superoxide anion,^[^ ^16^, ^17^ ^]^ the ability of DEF‐OCNs to increase lactate generation and glycolytic flux in cell and brain tissue may be another mechanism by which they confer cell and tissue‐level protection in hemorrhagic TBI (Figure 9). Dysfunctional mitochondrial respiration as a result of toxic metabolite accumulation in hemorrhagic TBI is believed to inhibit cellular recovery following the primary lesion and increase the demand for glycolytic energy metabolism.^[^ ^1^, ^62^ ^]^ Indeed, increased cerebral glycolysis has been shown to be associated with improved long‐term recovery following subarachnoid hemorrhage in humans.^[^ ^67^ ^]^ Further, the direct intravenous supplementation of sodium lactate in patients with severe TBI was shown to increase cerebral pyruvate and glucose while decreasing glutamate and intracranial pressure, demonstrating that lactate mediates protection against metabolic dysregulation by increasing energetic flexibility.^[^ ^68^ ^]^ We thus predict that the enhancement of lactate generation by OCNs is a protective process that supports cell and tissue resilience to hemorrhagic trauma beyond that of a chelator, antioxidant, or promoter of glycolytic metabolism alone.

Also, the increased glycolytic flux that forms the basis of heightened lactate generation with OCN treatment may also serve a protective, homeostatic role in the context of hemorrhagic TBI. In particular, increased glycolytic activity in macrophages following intracerebral hemorrhage is necessary for the production of PGE_2_, a potent mediator of inflammatory resolution and wound healing.^[^ ^69^ ^]^ This underscores the fact that glycolysis forms a nexus between energy metabolism and other pathways such as nucleotide synthesis and inflammatory signaling that are intricately linked with cellular homeostasis.^[^ ^70^ ^]^ This includes metabolic routes such as the pentose phosphate pathway (PPP) that is critical in regenerating NADPH, which is essential for maintaining cellular defense against oxidative damage.^[^ ^71^ ^]^ An important limitation to this study's usage of extracellular flux analysis to investigate metabolic changes is the difficulty of ascertaining the contributions of glycolytic feeder pathways such as the PPP and glycolytically‐coupled processes such as inflammatory signaling and ROS homeostatic mechanisms on total cellular energy metabolism. Thus, future experiments are needed to pursue targeted investigations of OCNs’ catalytic role in these individual pathways in order to elucidate their therapeutic relevance in other disease models.

## 4 Experimental Section

### Reagents

Powdered medical‐grade activated charcoal was purchased from EnviroSupply & Service (Dallas, TX, USA). Fuming HNO_3_ (90%, ACS grade) was purchased from Alfa Aesar (Haverhill, MA, USA). Iron(III) nitrate nonahydrate was purchased from Fisher Scientific (Hampton, NH, USA). *N,N*‐Dimethylformamide (DMF, ≥99.9%, HPLC grade), nitrilotriacetic acid disodium salt, and phosphate‐buffered saline (PBS, pH 7.4) were purchased from MilliporeSigma (Burlington, MA, USA). Methoxy‐poly(ethyleneglycol)‐amine (mPEG‐NH_2_), with an average molecular weight of 5000 Da, was purchased from Laysan Bio, Inc (Arab, AL, USA). *N,N*‐diisopropylcarbodiimide (DIC, >98.0%) was purchased from Tokyo Chemical Industry (TCI America, Portland, OR, USA).

### Cell Lines

bEnd.3 murine endothelioma cells (CRL‐2299) were purchased from the American Type Culture Collection (ATCC, Manassas, VA, USA). Primary human dermal fibroblasts (GM03440) from an Apparently Healthy Individual were obtained from the Coriell Institute for Medical Research (Camden, NJ, USA).

### Cell Culture

bEnd.3 cells were cultured in Dulbecco's Modified Eagle's Media (DMEM) with high glucose (Gibco, Waltham, MA, USA), 10% FBS (Atlanta Biologicals, Flowery Branch, GA, USA), and 1% (10 000 U mL^−1^) penicillin‐streptomycin (Lonza USA, Walkersville, MD, USA). Fibroblasts were cultured in DMEM media with high glucose (Gibco) 15% non‐heat inactivated FBS (Atlanta Biologicals), and 1% (10 000 U mL^−1^) penicillin‐streptomycin. Both types of cells were cultured in T‐75 filter vented flasks at 37 °C with 5% CO_2_.

### Animals

All experimental procedures were conducted in accordance with the Guide for the Care and Use of Laboratory Animals of the National Institutes of Health and were approved by the Institutional Animal Care and Use Committee (IACUC) using procedures consistent with those outlined in the ARRIVE guidelines. Male Sprague–Dawley rats (275–300 g) were purchased from Envigo (Houston, TX, USA). Rats were group‐housed on a 12‐h light/dark cycle, with ad libitum access to food and water. All experiments were performed during the light cycle.

### Controlled Cortical Impact Injury

Surgical procedures were approved by the Institutional Animal Care and Use Committee and were conducted in accordance with the recommendations provided in the Guide for the Care and Use of Laboratory Animals. Protocols were designed to minimize pain and discomfort during the injury procedure and recovery period.^[^ ^38^ ^]^ An electromagnet‐driven controlled cortical impact (CCI) injury device (Leica Biosystems, Richmond, IL, USA) was used to cause brain injury as previously described.^[^ ^37^ ^]^ Protocols were designed to minimize pain and discomfort during the injury procedure and recovery period.^[^ ^38^ ^]^ An electromagnet‐driven controlled cortical impact (CCI) injury device (Leica Biosystems, Richmond, IL) was used to cause brain injury as previously described.^[^ ^37^ ^]^ Briefly, animals were anesthetized using 5% isoflurane with a 1:1 O_2_/N_2_O mixture and mounted on a stereotaxic frame using both incisor and ear bars while anesthesia was maintained throughout the surgery with 2.5% isoflurane in a 1:1 mixture of O_2_/air administered by a face mask. A midline incision was made and a unilateral craniectomy (6 mm diameter) on the right side was produced midway between the bregma and lambda with the medial edge of the craniectomy 0.5 mm lateral to the midline. A single impact with 5 m s^−1^ velocity and 2.0 mm deformation was delivered on the right parietal lobe using a 5 mm diameter impactor tip. Core body temperature was maintained at 37 °C using a heating pad coupled with a rectal thermometer. Sham animals received all the surgical procedures except craniectomy and injury. Recovery of pain reflexes and restoration of the righting response were recorded immediately after surgery to ascertain consistency in the injury. Nanozyme (2 mg kg^−1^) or vehicle control (volume equivalent of saline) was administered by intravenous injection at 15 min post‐injury, and then by intraperitoneal injection at 2 hrs and 22 hrs after injury. Brain tissue punches (1.0 mm in diameter) were prepared from the cortical region 1 mm lateral to the contusion core 24 hrs after injury.

### Preparation of Brain Slices

Rats were humanely euthanized, brains were rapidly removed (within 30 s of decapitation) and immersed in ice‐cold (4–5 °C) artificial cerebrospinal fluid (aCSF; 120 mm NaCl, 3.5 mm KCl, 1.3 mm CaCl_2_, 1 mm MgCl_2_, 0.4 mm KH_2_PO_4_, 5 mm HEPES, and 10 mm D‐glucose; pH 7.4) that had been oxygenated for 1 h using 95% O_2_:5% CO_2_. Coronal sections (200 µm) were prepared using a modified McIlwain tissue chopper (Ted Pella. Inc.; Redding, CA, USA) with a chilled stage and blade, then transferred to a holding chamber containing continuously oxygenated aCSF at room temperature (≈23 °C).

### Immunofluorescence Analysis of Astrocyte Activation

Tissue samples from all groups were frozen and sectioned at a thickness of ≈45 µm using a Leica cryostat (Leica Biosystems, Milton Keynes, United Kingdom). Free‐floating tissue sections were incubated overnight with a primary antibody against glial fibrillary acidic protein (GFAP, 1:500 dilution, Abcam #ab7260), a marker of astrogliosis activation. Sections were then stained with a goat anti‐rabbit secondary antibody (*λ*_em:_488 nm, 1:1000 dilution, Fisher Scientific). As a nuclear counterstain, sections were incubated with 4′,6‐diamidino‐2‐phenylindole (DAPI; 1:10000 dilution, Fisher Scientific) for 5 min. Fluorescence signals were visualized using an Olympus Confocal Microscope FV3000 (Shinjuku City, Tokyo, Japan).

### Extracellular Flux Assays – Cells

Prior to each assay, each well of an Agilent Seahorse XFe96 plate was seeded with 20 000 bEnd.3 (murine brain endothelial) cells (ATCC, #CRL‐2299) in Dulbecco's Modified Eagle Medium (DMEM) (Thermo Fisher Scientific, #11965092). PEG‐HCC or methylene blue (MB) treatment conditions were administered to the appropriate wells, and the plate was incubated at 37 °C with 5% CO_2_ overnight. The accompanying sensor cartridge was hydrated with XF Calibrant (Agilent Technologies, Santa Rosa, CA, #100840‐000) at 37 °C under normal atmospheric conditions overnight. On the day of the assay, the culture medium was removed and 180 µL XF DMEM medium (Agilent Technologies, #103575‐100) with 1 mm pyruvate, 2 mm glutamine, and 10 mm glucose was added to each well. On the day of the assay, culture growth media was removed and 180 µL XF DMEM media (Agilent Technologies, #103575‐100) with 1 mm pyruvate, 2 mm glutamine, and 10 mm glucose was added to each well. For each assay type, the sensor cartridge was loaded with the following reagents:

Mitochondrial Stress (Agilent Technologies, #103015‐100) – oligomycin,1 µm, Carbonyl cyanide‐4 (trifluoromethoxy) phenylhydrazone FCCP; 1 µm, rotenone and antimycin A (0.75 µm).

ATP Rate (Agilent Technologies, #1003592‐100) – Oligomycin 1.5 µm, Rotenone/Antimycin A (0.5 µm)

Glycolytic Rate (Agilent Technologies, #103344‐100) – Rotenone/Antimycin A (0.5 µm), 2‐deoxy‐D‐glucose (50 mm)

Oxygen consumption rate (OCR) and extracellular acidification rate (ECAR) were measured using an Agilent Seahorse XFe96 Extracellular Flux Analyzer.

OCR and ECAR data from ATP Rate and Glycolytic Rate assays were used to quantitatively determine mitochondrial and glycolytic ATP production rates as well as basal and compensatory parameters of glycolytic rate.^[^ ^25^, ^72^ ^]^

### Extracellular Flux Assays — Brain Tissue Respiration

Brain sections were individually transferred to a biopsy chamber containing fresh oxygenated aCSF. A stainless steel WellTech Rapid‐Core biopsy punch needle (500 µm diameter; World Precision Instruments; Sarasota, FL) was used to excise the tissue punches. Tissue punches were taken from each anatomical location using four consecutive coronal sections (i.e., a total of 4 punches for each anatomical structure) using the same biopsy punch needle. Punches were ejected directly into an XFe96 Cell Culture Microplate (Agilent Technologies, #101085‐004) based on a pre‐ determined plate layout. Each well contained 180 µL room temperature assay media (aCSF supplemented with 0.6 mm pyruvate and 4 mg mL^−1^ lyophilized BSA). After loading all biopsy samples, each well was visually inspected to ensure that the punch was submerged and centered at the bottom. The XFe96 Cell Culture Microplate was then incubated at 37 °C for ≈30 min. During this incubation period, 10 × concentration of assay drugs (prepared in aCSF) were loaded into their respective injection ports of a hydrated (overnight in distilled water, exchanged for XF Calibrant solution 3 h prior to assay initiation) Seahorse XFe96 Extracellular Flux Assay sensor cartridge. The sensor cartridge containing the study drugs was then inserted into the analyzer for calibration. Once the analyzer was calibrated, the calibration plate was replaced by the microplate containing the tissue punches, and the assay protocol was initiated. Assay drugs were prepared at 10 × working concentrations in oxygenated aCSF (pH 7.4) and delivered sequentially to achieve final concentrations of: port A: Oligomycin (25 µg mL^−1^); port B: FCCP + pyruvate (75 µm and 7.5 mm, respectively); and port C: Antimycin A + rotenone (10 µm and 5 µm, respectively). The dose of each of these reagents was based on optimization experiments to achieve optimum drug effect.^[^ ^36^ ^]^ The duration of sampling time (for calculating the oxygen consumption rate or OCR) for each condition was determined to allow the effect of each drug to reach a steady state.

### Extracellular Lactate Measurement

Agilent Seahorse XFe96 plates were seeded with 20 000 bEnd.3 cells/well, with culture media exchanged to phenol red‐free DMEM (Gibco, #21063029). Plates were incubated overnight with DEF‐PEG‐cOACs, PEG‐OACs, PEG‐HCCs, or PBS at 37 °C with 5% CO_2_, and media from each well was decanted and assayed for lactate concentration using an NADH‐independent colorimetric assay (Cell Biolabs, #MET‐5012) as per manufacturer's instructions.

### Synthesis of Poly(ethylene glycol)‐functionalized Hydrophilic Carbon Clusters (PEG‐HCCs)

Synthesis and functionalization of poly(ethylene glycol)‐functionalized hydrophilic carbon clusters (PEG‐HCCs) was developed in the previous publication.^[^ ^4^, ^16^, ^73^ ^]^ HCCs were graphene nanoribbons that were ≈3 × 40 nm and were synthesized by the longitudinal oxidative splitting of single‐walled carbon nanotubes (SWCNTs). Briefly, HCCs were prepared by oxidizing single‐walled carbon nanotubes using a mixture of oleum and nitric acid. To HCCs, PEG groups were attached via a carbodiimide coupling reaction.^[^ ^16^, ^73^ ^]^

An aqueous solution of PEG‐HCCs (5.0 mL, 1.2 mg mL^−1^, 6 mg) was added to ethylenediamine (5.0 mL, 4.5 g, 75 mmol). Water was removed via rotovap, leading to the precipitation of the PEG‐HCCs in ethylenediamine. Methanol (6 mL) was added to disperse the PEG‐HCCs, along with molecular sieves. The reaction was allowed to stir at room temperature for 5 d before dilution in deionized H_2_O and filtration using a 0.22 µm polyethersulfone (PES) membrane. The material was purified via crossflow filtration (Spectrum Labs Krosflo, Research IIi TFF System) with a 50 kDa mPES dialysis column (≈1 atm transmembrane pressure) to yield 15 mL of PEG‐HCC with a carbon core concentration of 400 mg L^−1^.^[^ ^4^ ^]^ The concentration of PEG‐HCCs in solution was estimated based on the carbon core absorbance at 763 nm using an extinction coefficient of 0.01040 L mg^−1^ or 0.00428 nm ^−1^.^[^ ^4^, ^16^ ^]^

### Synthesis of Coconut‐derived Oxidized Activated Charcoal Nanozymes (cOACs)

It was previously reported the synthesis of cOACs by fuming nitric acid‐mediated oxidation.^[^ ^64^, ^74^ ^]^ Powdered medical grade coconut‐derived activated charcoal (cAC; 0.506 g, 42.2 mmol carbon) was added to a 250 mL round‐bottom flask, followed by the addition of fuming (90%) HNO_3_ (50 mL, 1.2 mol). The reaction mixture was placed in an oil bath pre‐heated to 100 °C and was allowed to stir under reflux for 4 or 6 h. After heating, the reaction mixture was removed from the heat source and cooled to room temperature. The reaction was quenched by pouring the contents of the round‐bottom flask over 70 mL of deionized (DI) ice in a 2 L beaker. The quenched solution was allowed to reach room temperature before pouring into one Spectra/Por 7 dialysis membrane (regenerated cellulose, 1 kDa MWCO, 40 cm x 45 mm). The resulting cOAC solution was purified via bath dialysis over the course of 7 days, subsequently filtered through a 0.22 µm PES membrane, and dried by lyophilization (Labconco Freeze Dry System/Freezone 4.5).

Synthesis of Poly(Ethylene Glycol)‐Functionalized cOACs (PEG‐cOACs) and Deferoxamine‐Conjugated PEG‐cOACs (DEF‐PEG‐cOACs): The lyophilized cOAC (35.8 mg, 2.98 mmol) was suspended in DMF (30 mL) and cup horn sonicated for 60 min at 50% amplitude (Cole‐Parmer Ultrasonic Processor CP 750). For PEG‐cOAC synthesis, 5 kDa methoxy‐PEG‐amine (0.3588 g, 0.07176 mmol) was added to the reaction vessel. For DEF‐PEG‐cOAC synthesis, deferoxamine mesylate salt (20.6 mg, 0.0314 mmol) and *N,N*‐diisopropylethylamine (0.027 mL, 0.16 mmol) were added to the reaction vessel, followed by 5 kDa methoxy‐PEG‐amine (0.1705 g, 0.03410 mmol). The mixture was bath‐sonicated for 20 min (Cole‐Parmer Cat #08849‐00). The reaction vessel was removed from the sonicator and DIC (0.358 mL, 2.32 mmol) was added. The solution was stirred for 48 h at room temperature. Prior to purification by bath dialysis, excess DMF was removed from the reaction mixture by centrifugation (NuAire NU‐C200R, parameters: 4500 g, 30 min, room temperature) using Amicon Ultra‐15 Centrifugal Filters (regenerated cellulose, 10 kDa MWCO). The resulting PEG‐cOAC‐containing retentate was diluted in Milli‐Q water and then transferred to one Float‐A‐Lyzer G2 dialysis device (regenerated cellulose, MWCO 50 kDa, 10 mL volume capacity) for purification via dialysis. The filled device was placed in a 2 L beaker containing Milli‐Q®‐purified water (MilliporeSigma) and continuously stirred by a magnetic stir bar/stir plate for 48 h. The water bath was exchanged 6–8 times in this interval. After dialysis, the PEG‐cOAC or DEF‐PEG‐cOAC particles were sterile‐filtered using a 0.22 µm PES membrane and dissolved in PBS (pH 7.4). UV–Vis spectrophotometry was used to determine the cOAC carbon core concentration of the aqueous PEG‐cOAC and DEF‐PEG‐cOAC products (mass extinction coefficient 3.48 mg^−1^·mL^−1^, λ = 700 nm).

### Data Analysis, Extracellular Flux Analyses

All computational analyses were performed using R 4.2.2.^[^ ^75^ ^]^ OCR and ECAR data from each extracellular flux assay were aggregated by well and assay timepoint, and wells with consistent outlier data values across multiple assay timepoints (“well‐effect outliers”) were identified using the OCR‐stats algorithm (https://github.com/gagneurlab/OCR‐Stats).^[^ ^76^ ^]^ OCR stats were independently applied to OCR and ECAR data to generate two sets of well‐effect outliers, which were merged to form a complete set of wells that were subsequently excluded from further analysis. All bioenergetic parameters were calculated using Agilent Seahorse XF default specifications for mitochondrial stress, glycolytic rate, and ATP production rate assays respectively.^[^ ^30^, ^72^, ^77^, ^78^ ^]^ Comparisons of extracellular flux and lactate assay parameters for treatment conditions across multiple assays were performed using paired t‐tests (for two‐group comparisons) or repeated‐measures ANOVA with paired t‐test post‐hoc analyses. All statistical analyses were performed using the R *stats* ^[^ ^75^ ^]^ and *rstatix* ^[^ ^79^ ^]^ libraries. Results were visualized using the *ggplot2* library^[^ ^80^ ^]^ and were presented as mean + SEM.

### Data Analysis, Colorimetric Image Quantification

2D color images containing the superior surfaces of whole rat brains were captured using a Leica S9i Digital Stereo Microscope (Leica GmbH; Wetzlar, Germany). Images were projected onto the CIELAB space, a representation of color as a combination of lightness (*L* value), red/green intensity (a* value), and blue/yellow intensity (b* value).^[^ ^81^ ^]^ Binary masks corresponding to the approximate area of hemorrhage on the surface of each brain were generated from the a* values of each image using ImageJ (NIH; Bethesda, MD). The area was calculated by converting the total pixel area of each mask to mm^2^ using the average pixel length from 5 tick spaces of a 1 mm‐increment scale bar. Pixel intensity information was retrieved from each image by using its respective Hue‐Saturation‐Brightness (HSB) projection and inverting values from the “brightness” channel to obtain a quantitative metric of darkness due to hemorrhage.^[^ ^82^ ^]^

### Intracellular Free Iron Measurement

Murine brain endothelial (bEnd.3) cells were seeded onto a 24‐well plate at a density of 20 000 cells mL^−1^ and cultured overnight in the presence of 4 µg mL^−1^ DEF‐PEG‐cOACs or 50 µm deferoxamine mesylate (Thermo Fisher Scientific, #461770010) at 37 °C with 5% CO_2_. Cells were rinsed with phosphate‐buffered saline (PBS) and incubated with 1 µm FerroOrange (Dojindo Molecular Technologies, #F374) and 50 µm iron (III) chloride (Sigma‐Aldrich, #157740) at 37 °C for 30 min. Cells were rinsed with PBS 3 × and visualized following the manufacturer's instructions using fluorescence microscopy (Leica Microsystems DMi8). Intracellular regions of fluorescent intensity were identified using ImageJ (NIH, Bethesda, MD) and its implementations of the Otsu method^[^ ^35^ ^]^ and feature‐based segmentation algorithms. Identified regions of interest were filtered using size exclusion thresholds to exclude overconfluent cell regions and debris, and the resulting selections were used to compute intracellular mean fluorescence intensities from raw images.

## Conflict of Interest

The universities own intellectual property (IP) on the carbon nanoparticles described here. That IP is being licensed to companies in which J.M.T., P.J.D., and T.A.K. are shareholders. J.M.T. and P.J.D. are not officers, directors, or employees of those companies. T.A.K. is an officer in Gerenox Inc. Conflicts of interest for J.M.T. are mitigated through regular disclosure and compliance with the Rice University Office of Sponsored Programs and Research Compliance. Conflicts of interest for P.J.D. and T.A.K. are mitigated through regular disclosure to and compliance with the Texas A&M University Office of Sponsored Programs.

## Supporting information



Supporting Information

## Data Availability

The data that support the findings of this study are available from the corresponding author upon reasonable request.
