# Supplementary material for: Oxidized Carbon Nanoparticles Enhance Cellular Energetics With Application to Injured Brain
Source: Adv Healthc Mater. 2024 Sep 27;14(8):2401629. doi: 10.1002/adhm.202401629 (PMC11937864; doi:10.1002/adhm.202401629)
Supplement: Supplementary file 1 — Supporting Information [file ADHM-14-0-s001.pdf]

ADVANCED HEALTHCARE MATERIALS

Supporting Information

for *Adv. Healthcare Mater.*, DOI 10.1002/adhm.202401629

Oxidized Carbon Nanoparticles Enhance Cellular Energetics With Application to Injured Brain

Karthik Mouli, Anton V. Liopo, Emily A. McHugh, Erica Underwood, Jing Zhao, Pramod K. Dash, Anh T. T. Vo, Vikas H. Malojirao, Muralidhar L. Hegde, James M. Tour, Paul J. Derry and Thomas A. Kent**

Supplementary Information:

Methods:

Histological staining of brain tissue sections: Paraformaldehyde-fixed brain tissues were embedded in paraffin and sectioned into 5 µm slices using a Leica Histocore Biocut rotary microtome (Leica Biosystems, Milton Keynes, United Kingdom) to ensure uniform thickness. The slides were baked in an incubator at 60°C for 2 hours. The slices were then de-paraffinized in xylene twice for 5 minutes each, followed by rinsing in 100%, 95%, 80%, and 75% ethanol solutions for 1 minute each. Finally, they were rinsed in distilled water for 2 minutes at room temperature. Subsequently, some slices were stained using a commercial hematoxylin/eosin staining kit (Abcam, ab245880, Cambridge, MA, USA) following the manufacturer's standard procedures. The hematoxylin and eosin-stained slides were then scanned with an Aperio CS2 scanner (Leica Biosystems).

Figure S1:

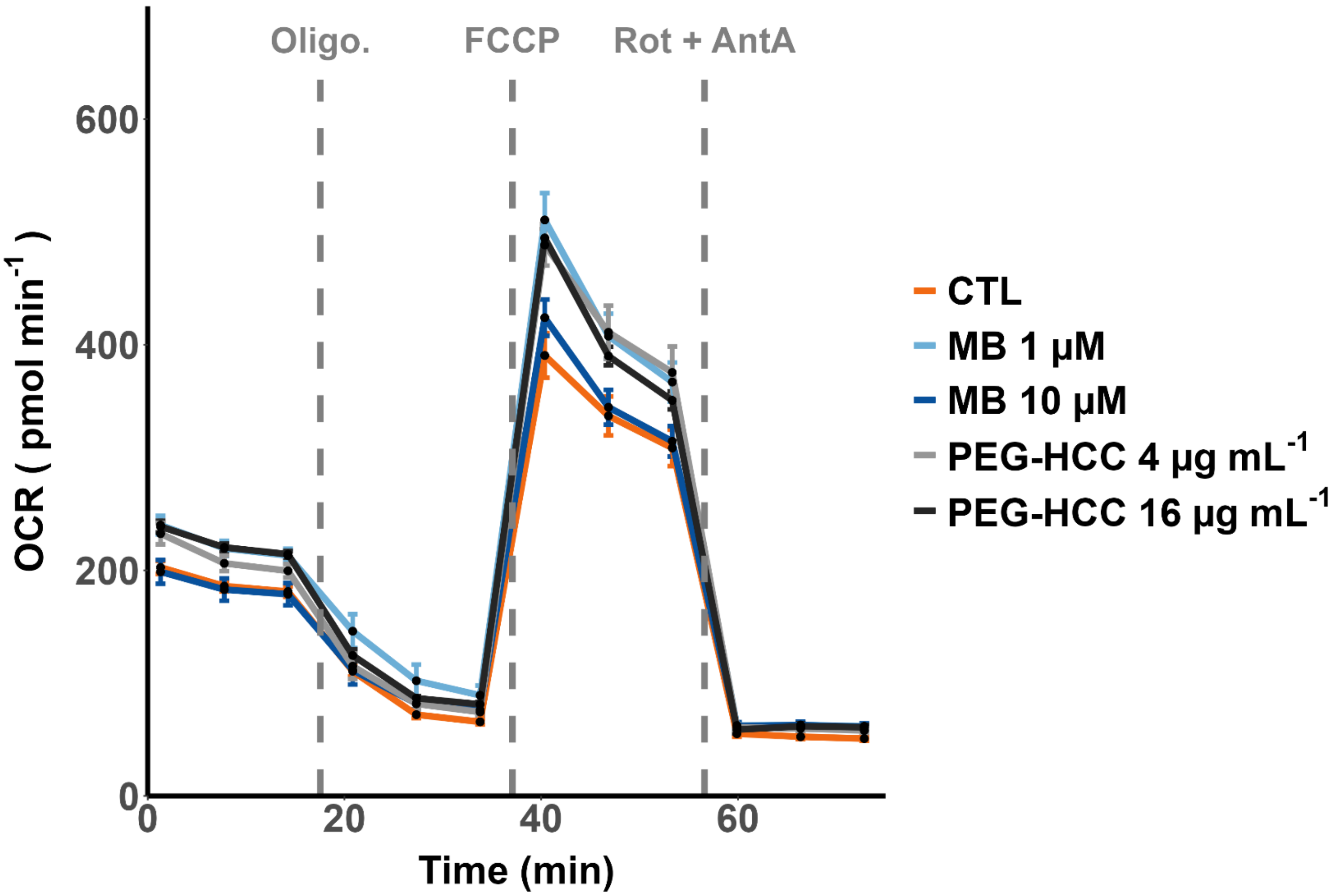

Figure S1: Oxygen consumption rate (OCR) in bEnd.3 brain endothelial cells pretreated with PEGylated hydrophilic carbon clusters (PEG-HCC) and methylene blue (MB) relative to cells treated with phosphate-buffered saline (CTL) initially and after the addition of oligomycin and FCCP respectively. OCR is increased with both PEG-HCC doses, but there is decreased basal and maximal OCR with 10 μM MB relative to 1 μM MB that may indicate an oversaturation of mitochondrial oxidative phosphorylation at higher doses. Dashed lines indicate the approximate timepoint of oligomycin (Oligo.), FCCP and rotenone/antimycin A (Rot + AntA) addition. Mean + SEM, one-way ANOVA; * $p < 0.05$; ** $p < 0.01$; *** $p < 0.001$.

Figure S2:

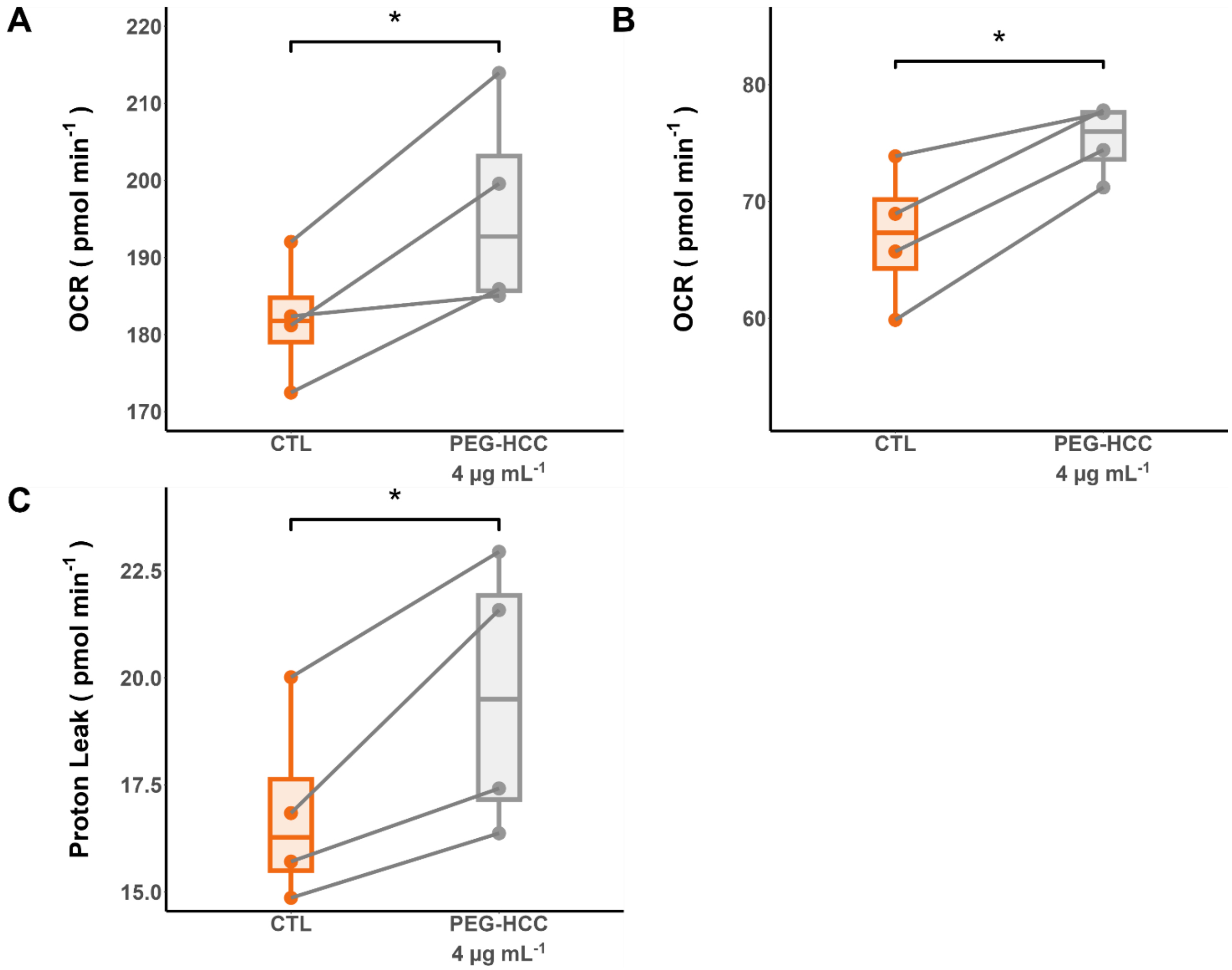

Figure S2**:** Individual bioenergetic parameters, demonstrating increased oxygen consumption rate (OCR) in bEnd.3 brain endothelial cells pretreated with 4 μg mL^{-1} PEGylated hydrophilic carbon clusters (PEG-HCC) (A) prior to the addition of mitochondrial respiratory inhibitors and (B) after the addition of 1 μM oligomycin. (C) Higher proton leak in bEnd.3 cells pretreated with 4 μg mL^{-1} PEG-HCCs. Proton leak is calculated as the difference between the minimum OCR value following oligomycin administration and the minimum OCR value after the addition of rotenone and antimycin A. Higher proton leak values signify a greater extent of ATP-uncoupled proton transport across the inner mitochondrial membrane, a mechanism of mitochondrial protection against reactive oxygen species generation. Persistence of higher

OCR in the presence of oligomycin with PEG-HCC treatment may indicate that PEG-HCCs buffer mitochondrial respiration against metabolic insults. Each dot represents a different mitochondrial stress assay, with lines connecting groups from the same assay. Boxplots depict mean + SEM. Paired t-tests; * $p < 0.05$; ** $p < 0.01$; *** $p < 0.001$.

Figure S3:

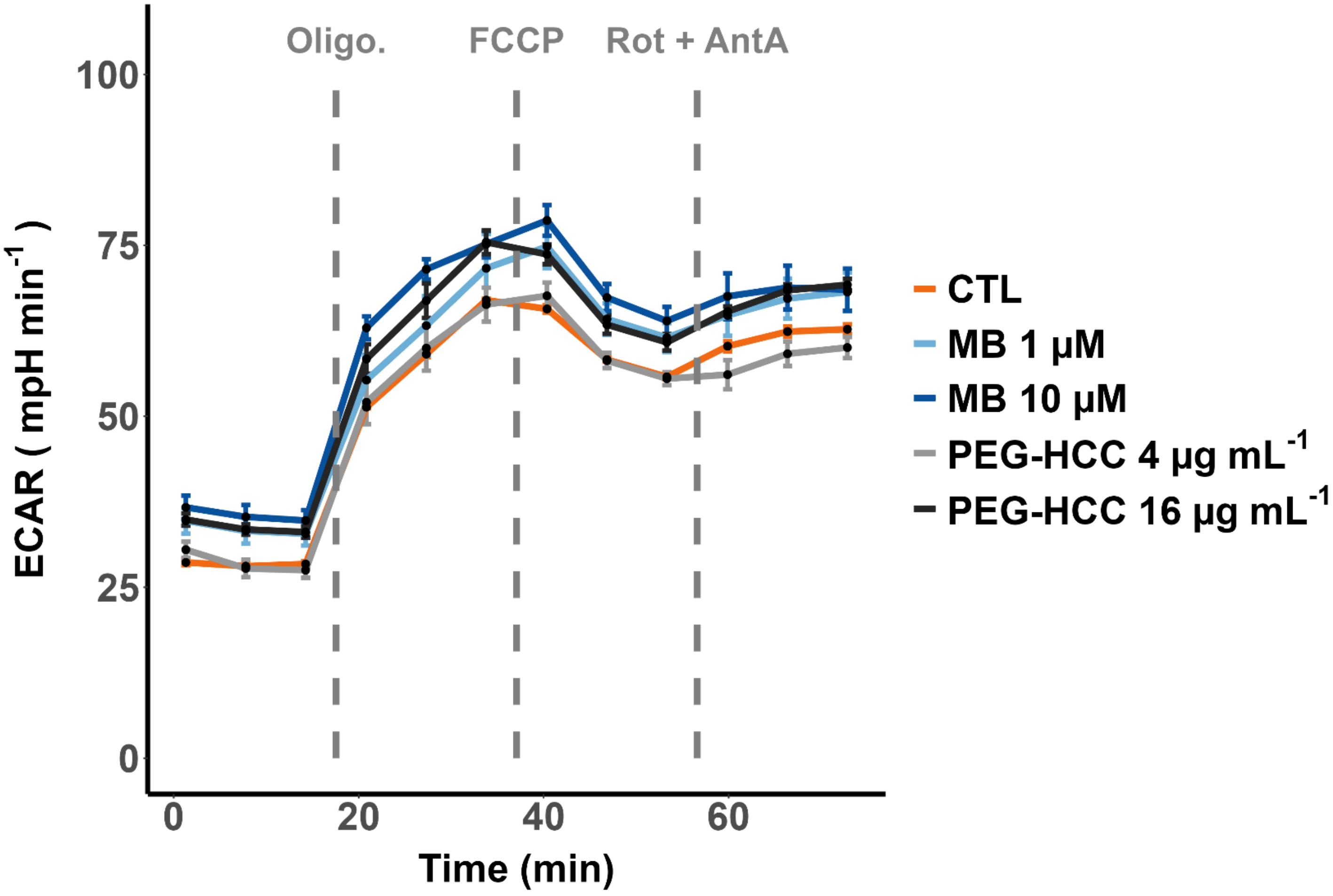

Figure S3: Higher extracellular acidification rate (ECAR) in bEnd.3 brain endothelial cells pretreated with PEGylated hydrophilic carbon clusters (PEG-HCC) at 16 µg mL^{-1} as well as 10 µM methylene blue (MB). No significant difference in assay-wide ECAR between 1 and 10 µM MB, which may indicate oversaturation of glycolytic flux at higher effector doses. Dashed lines indicate the approximate timepoint of oligomycin (Oligo.), FCCP and rotenone/antimycin A (Rot + AntA) addition. Mean + SEM, one-way ANOVA; * $p < 0.05$; ** $p < 0.01$; *** $p < 0.001$.

Figure S4:

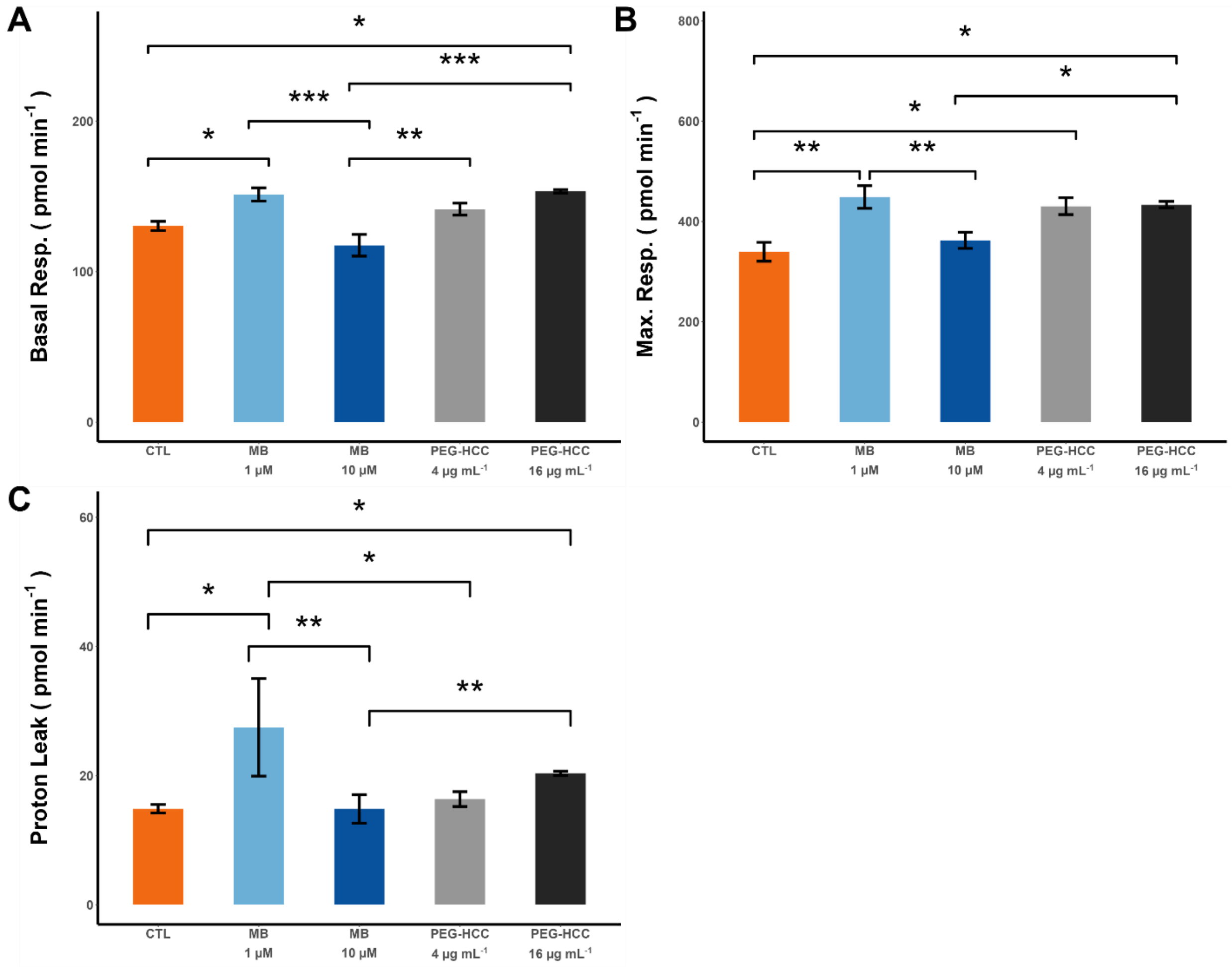

Figure S4: Higher (A) basal respiration rate with PEGylated hydrophilic carbon clusters (PEG-HCC) at 16 μg mL^{-1} and 1 μM methylene blue (MB), (B) maximum respiration rate with both PEG-HCC doses and 1 μM MB and (C) proton leak with 16 μg mL^{-1} PEG-HCC and 1 μM MB treatment relative to cells treated with PBS (CTL). Basal respiration is calculated as the difference between initial baseline and minimum oxygen consumption rates (OCR) following the addition of rotenone and antimycin A. Maximum respiration is calculated as the difference between the maximum OCR value following FCCP addition and the minimum OCR following the addition of rotenone and antimycin A. Proton leak is calculated as the difference between the minimum OCR value following oligomycin administration and the minimum OCR value after the addition of rotenone and antimycin A. Decreased basal and maximal

respiration as well as proton leak with 10 μM MB relative to 1 μM may indicate an oversaturation of mitochondrial aerobic energy metabolism at higher MB doses. Mean + SEM, one-way ANOVA with Tukey's HSD post-hoc test; * $p < 0.05$; ** $p < 0.01$; *** $p < 0.001$.

Figure S5:

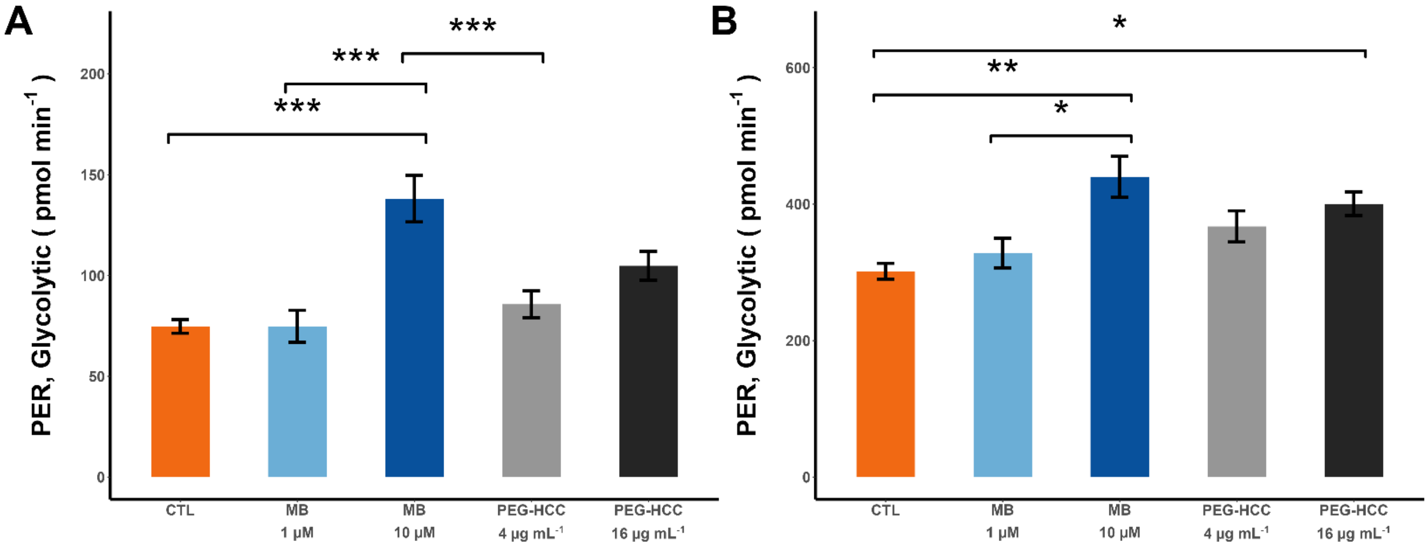

Figure S5: Higher (A) basal glycolytic rate (PER; proton efflux rate) with 10 µM methylene blue (MB) treatment only and (B) glycolytic rate after addition of rotenone and antimycin A with PEGylated hydrophilic carbon clusters (PEG-HCC) and 10 µM MB, relative to cells treated with PBS (CTL). A compensatory increase in glycolytic rate is a cytoprotective mechanism following the inhibition of mitochondrial energy metabolism, and an increase in the response with PEG-HCC treatment may indicate greater cellular resilience to mitochondrially-toxic stressors. Mean + SEM, one-way ANOVA with Tukey's HSD post-hoc test; * $p < 0.05$; ** $p < 0.01$; *** $p < 0.001$.

Figure S6:

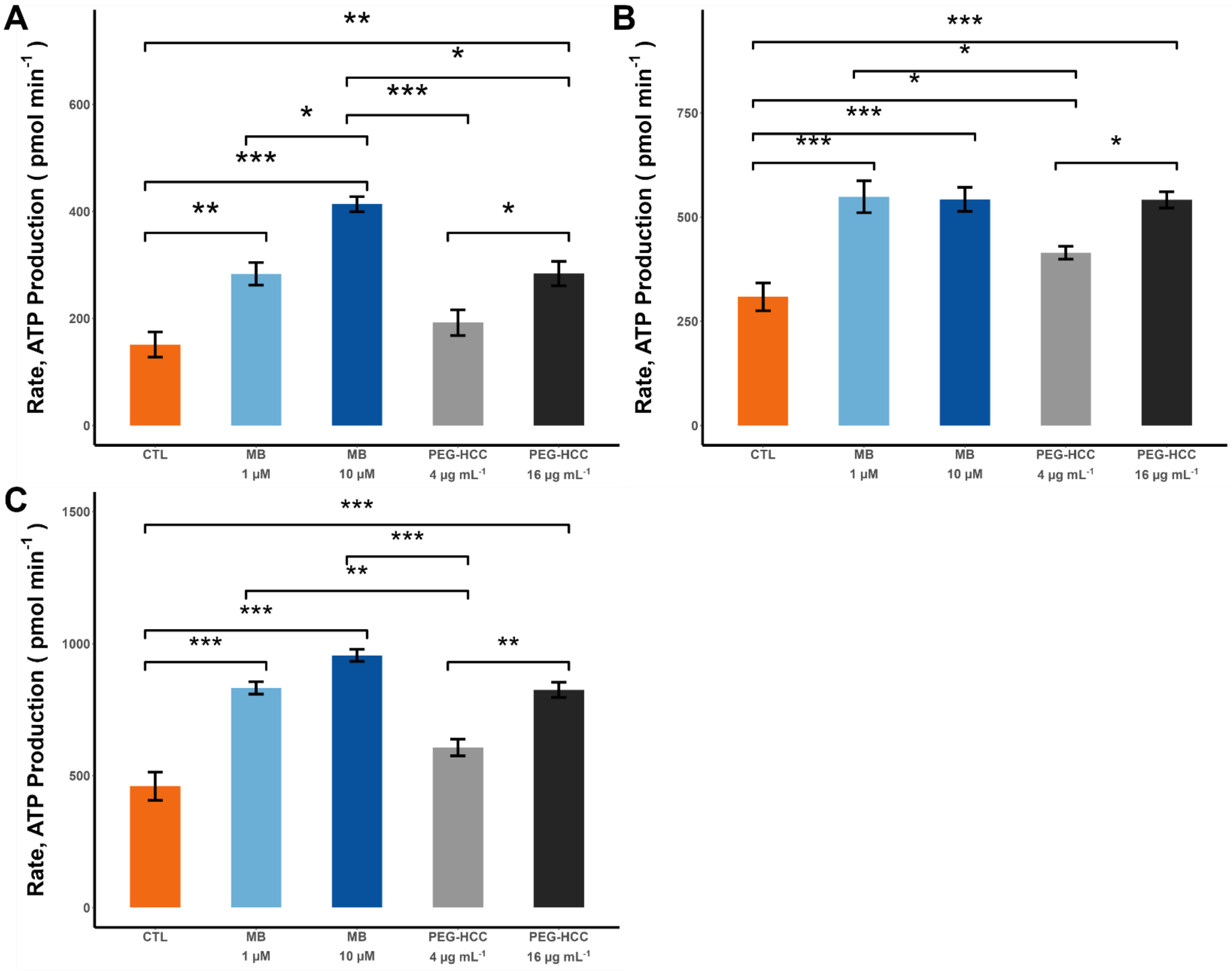

Figure S6: Higher (A) glycolytic ATP production rates with PEGylated hydrophilic carbon cluster (PEG-HCC) treatment at 16 µg mL^{-1} as well as with 1 and 10 µM methylene blue (MB), with dose-dependent increases observed with both effectors, (B) mitochondrial ATP production rates with both PEG-HCC and methylene blue treatment levels and (C) total ATP production rates with 16 µg mL^{-1} PEG-HCC and both methylene blue treatment groups, relative to cells treated with PBS. Findings are consistent with higher

observed glycolytic flux in a dose-dependent manner for MB and PEG-HCCs. Mean + SEM, one-way ANOVA with Tukey's HSD post-hoc test; * $p < 0.05$; ** $p < 0.01$; *** $p < 0.001$.

Figure S7:

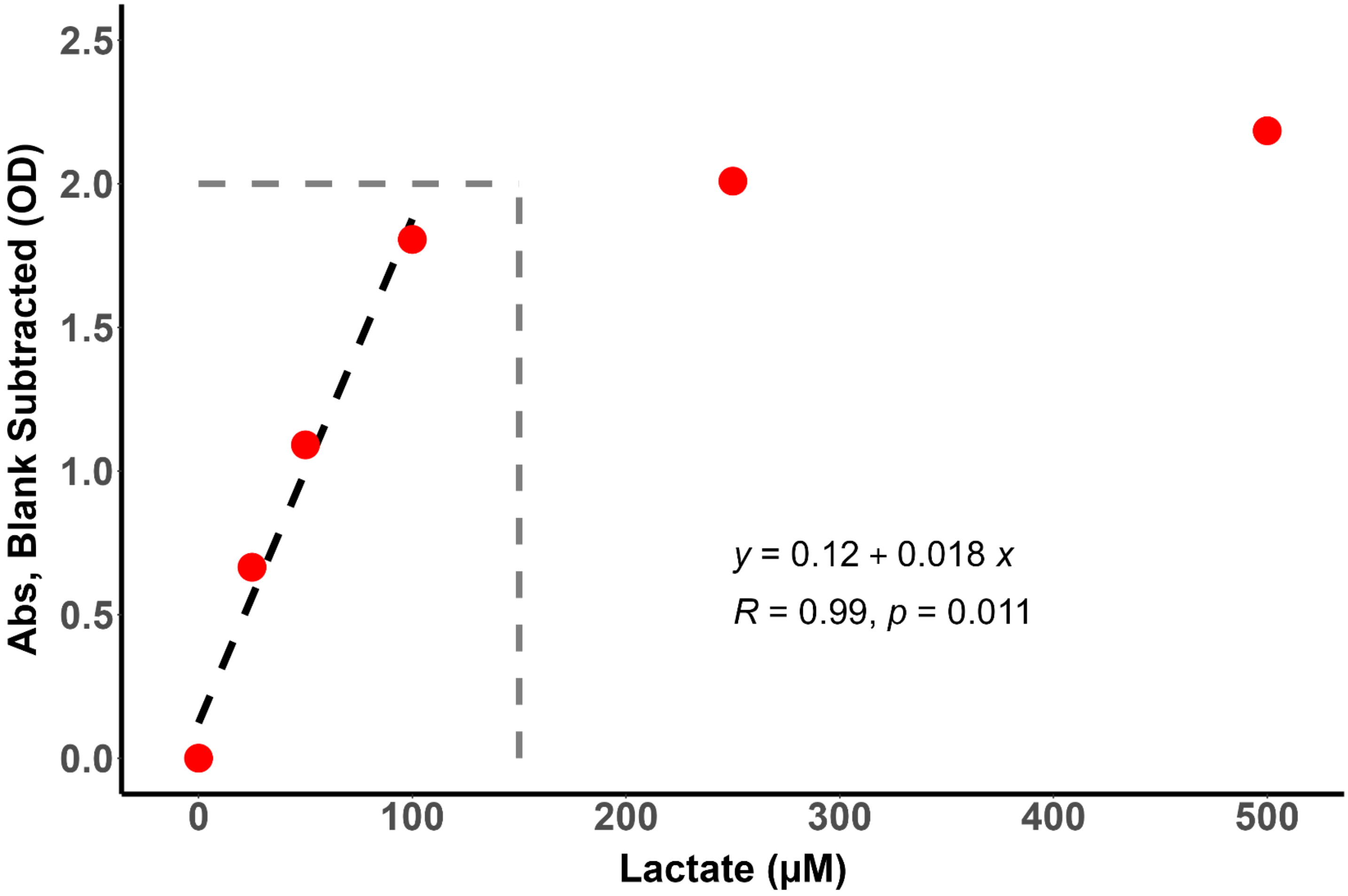

Figure S7: Standard curve using lactate (0-500 µM). Dashed enclosure represents the linear region of the curve, used for linear regression. Mean + SEM, Pearson's *r*.

Figure S8:

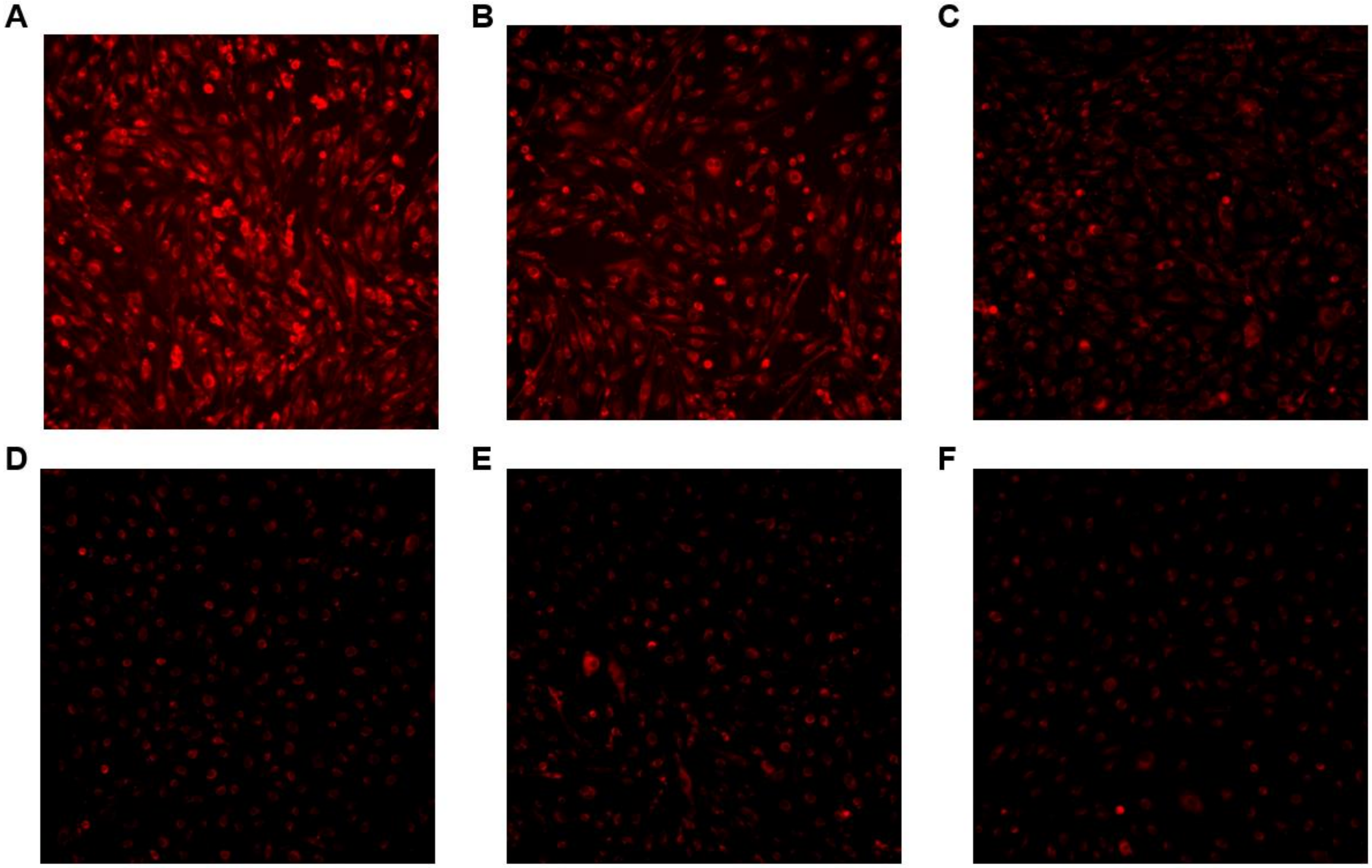

Figure S8: Measurement of intracellular free iron in brain endothelial (bEnd.3) cells using FerroOrange, a ferrous (Fe^{2+}) iron-specific fluorophore. Panels (A-C) show cells grown in Fe^{3+}-enriched media and treated with (A) no effectors, (B) 4 µg mL^{-1} DEF-PEG-cOACs or (C) 50 µM of the Fe^{3+} chelator deferoxamine mesylate for 24 hours prior to imaging via fluorescence microscopy. Panels (D-F) show cells grown in media without exogenous iron supplementation and treated with (A) no effectors, (B) 4 µg mL^{-1} DEF-PEG-cOACs or (C) 50 µM deferoxamine mesylate for 24 hours. In iron-supplemented cells, DEF-PEG-cOAC and deferoxamine treatment significantly decrease intracellular fluorescence, which supports the iron chelation activities of DEF-PEG-cOACs. No significant differences in fluorescence was observed between DEF-PEG-cOAC, deferoxamine and untreated control conditions in the absence of

media iron supplementation, indicating limited quenching or activation of FerroOrange fluorescence at these effector concentrations.

Figure S9:

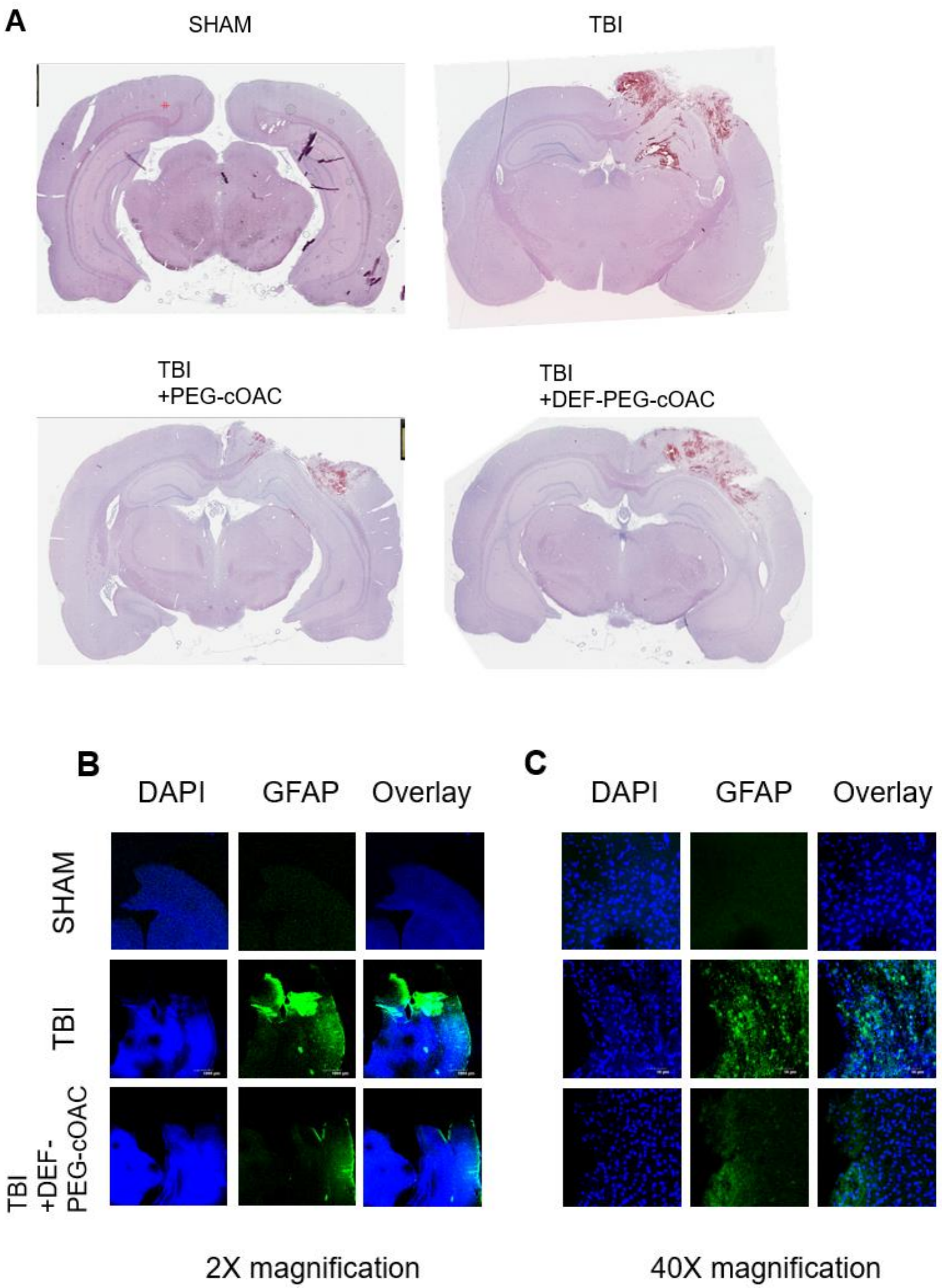

A
SHAM
TBI
TBI
+PEG-cOAC
TBI
+DEF-PEG-cOAC
B
DAPI
GFAP
Overlay
SHAM
TBI
TBI
+DEF-
PEG-cOAC
2X magnification
C
DAPI
GFAP
Overlay
40X magnification

Figure S9: Tissue-level effects of OCNs (PEG-cOACs, DEF-PEG-cOACs) in a rat model of contusion-based traumatic brain injury (TBI) with hemorrhage 3 days following injury (A) Hematoxylin/eosin histological staining of coronal brain sections from rats subject to TBI and treated with saline, PEG-cOACs and DEF-PEG-cOACs. Considerable tissue distortion is visible in the TBI with saline treatment. A large cortical defect without the tissue distortion is seen in the PEG-OAC treated rat. Decreased bleeding is present with a smaller cortical defect DEF-PEG-cOAC-treated rats. (B, C) Visualization of astrocyte activation within brain sections at (B) 2X and (C) 40X magnification. Green indicates positive glial fibrillary acidic protein (GFAP) staining, a marker of astrocyte activation, and blue fluorescence (DAPI) indicates cell nuclei. Sections from DEF-PEG-cOAC-treated rats subject to TBI exhibit decreased GFAP fluorescence relative to saline-treated (TBI) control. False positive GFAP staining is present in the considerable hematoma in the saline treated TBI example.
